# Amyloid-β-Driven Synaptic Deficits Are Mediated by Synaptic Removal of GluA3-Containing AMPA Receptors

**DOI:** 10.1523/JNEUROSCI.0393-24.2024

**Published:** 2025-01-08

**Authors:** Niels R. Reinders, Sophie J. F. van der Spek, Remco V. Klaassen, Karin J. Koymans, Harold D. MacGillavry, August B. Smit, Helmut W. Kessels

**Affiliations:** ^1^Netherlands Institute for Neuroscience, Royal Netherlands Academy of Arts and Sciences, Amsterdam 1105 BA, The Netherlands; ^2^Swammerdam Institute of Life Sciences, Amsterdam Neuroscience, University of Amsterdam, Amsterdam 1098 XH, The Netherlands; ^3^Center for Neurogenomics and Cognitive Research, Amsterdam Neuroscience, Vrije Universiteit Amsterdam, Amsterdam 1081 HV, The Netherlands; ^4^Division of Cell Biology, Neurobiology and Biophysics, Department of Biology, Faculty of Science, Utrecht University, Utrecht 3584 CH, The Netherlands

**Keywords:** Alzheimer, AMPA, amyloid, GluA3; PDZ domain, synapse

## Abstract

The detrimental effects of oligomeric amyloid-β (Aβ) on synapses are considered the leading cause for cognitive deficits in Alzheimer's disease. However, through which mechanism Aβ oligomers impair synaptic structure and function remains unknown. Here, we used electrophysiology and amino-3-hydroxy-5-methyl-4-isoxazolepropionic acid receptor (AMPAR) imaging on mouse and rat neurons to demonstrate that GluA3 expression in neurons lacking GluA3 is sufficient to resensitize their synapses to the damaging effects of Aβ, indicating that GluA3-containing AMPARs at synapses are necessary and sufficient for Aβ to induce synaptic deficits. We found that Aβ oligomers trigger the endocytosis of GluA3 and promote its translocation toward endolysosomal compartments for degradation. Mechanistically, these Aβ-driven effects critically depend on the PDZ-binding motif of GluA3. A single point mutation in the GluA3 PDZ-binding motif prevented Aβ-driven effects and rendered synapses fully resistant to the effects of Aβ. Correspondingly, proteomics on synaptosome fractions from APP/PS1-transgenic mice revealed a selective reduction of GluA3 at an early age. These findings support a model where the endocytosis and lysosomal degradation of GluA3-containing AMPARs are a critical early step in the cascade of events through which Aβ accumulation causes a loss of synapses.

## Significance Statement

Early cognitive symptoms in Alzheimer’s disease (AD) are considered to be driven by dysfunctional synapses. Studies in animal models for AD have demonstrated that the preservation of synapses alleviates cognitive symptoms. However, a clinically viable approach to preserve synapses requires a mechanistic understanding of how synaptic function is perturbed. Our study reveals that synapse impairments in Alzheimer models require the synaptic removal and subsequent lysosomal degradation of α-amino-3-hydroxy-5-methyl-4-isoxazolepropionic acid receptors that contain subunit GluA3. Preventing this aberrant trafficking of GluA3 leads to the preservation of synapses. This study identifies that the presence of GluA3 determines whether synapses are vulnerable to Alzheimer pathology, thereby deepening our understanding of how synapses are affected in AD.

## Introduction

Synapse loss is considered a major cause for cognitive deficits in Alzheimer's disease (AD) and is strongly correlated with AD symptoms (Selkoe, 2002; Targa Dias Anastacio et al., 2022). Studies in rodent models have shown that oligomeric amyloid-β (Aβ) induces the removal of amino-3-hydroxy-5-methyl-4-isoxazolepropionic acid receptors (AMPARs) from synapses leading to the loss of synapses (Hsieh et al., 2006; Guntupalli et al., 2016; Jurado, 2018). Aβ-mediated AMPAR removal from synapses is therefore considered to be a critical step in AD development.

The majority of AMPARs in CA1 pyramidal neurons consist of either subunits GluA1 and GluA2 (GluA1/2) or GluA2 and GluA3 (GluA2/3; Wenthold et al., 1996). The carboxy-terminal tail (C-tail) of GluA2 and GluA3 are similar in sequence and share an identical PDZ-binding motif. Through this motif, GluA2 and GluA3 can interact with PDZ-containing proteins glutamate receptor-interacting protein (GRIP) and PICK1. GRIP-binding controls the transport of AMPARs into dendrites and their stabilization at synapses (Osten et al., 2000; Setou et al., 2002; Heisler et al., 2014; Tan et al., 2015, 2020), whereas the interaction with PICK1 promotes AMPAR endocytosis and lysosomal degradation (Kim et al., 2001; Perez et al., 2001; Fiuza et al., 2017; Koszegi et al., 2017). The PDZ-binding motif can be phosphorylated by PKCα, which disrupts the interaction with GRIP but not with PICK1. As such, PKCα can regulate GRIP-/PICK1-mediated cycling of AMPARs in and out of synapses (Hanley, 2018; Moretto and Passafaro, 2018). Notably, PKCα-mediated phosphorylation and endocytosis of AMPARs through the interaction with PICK1 are involved in Aβ-driven synaptic depression and synapse loss (Hsieh et al., 2006; Alfonso et al., 2014, 2016).

Because PICK1 can interact with GluA2 and GluA3, both GluA1/2s and GluA2/3s are potentially susceptible to Aβ-mediated removal from synapses. However, we previously found that the expression of GluA3 is necessary for Aβ to trigger synaptic impairments, since neurons are fully resistant to Aβ-mediated synaptic depression when they lack GluA3 (Reinders et al., 2016). This observation raises the question whether GluA3-containing AMPARs are selectively targeted by Aβ and whether their presence is both necessary and sufficient for triggering synaptic deficits. We therefore set out to investigate how GluA3 influences the vulnerability of synapses for Aβ-driven synaptic depression. We expressed GluA3 or GluA3 mutants with altered PDZ-binding motifs in hippocampal neurons that either overproduced Aβ or were exposed to synthetic Aβ oligomers. Our experiments show that Aβ-mediated synaptic depression depends on protein interaction at the PDZ-binding motif of GluA3. Specifically, Aβ oligomers trigger a signaling cascade that drives the endocytosis and lysosomal degradation of GluA3-containing AMPARs leading to synaptic depression.

## Materials and Methods

### Animals

The GluA3-knock-out (KO) and wild-type littermate colony was established from C57Bl/6 × 129P2-Gria3tm1Dgen/Mmnc mutant ancestors (RRID:MMRRC_030969-UNC; MMRRC) and were at least 20 times backcrossed to C57Bl/6 mice. Mice were kept on a 12 h day/night cycle (light onset 8 or 7 A.M.) and had *ad libitum* access to food and water. Pregnant Wistar rats were directly obtained from Janvier Labs. All experiments were conducted in line with the European guidelines for the care and use of laboratory animals (Council Directive 86/6009/EEC). The experimental protocol was approved by the Animal Experiment Committee of the Royal Netherlands Academy of Arts and Sciences (KNAW), University of Amsterdam (UvA) and/or Utrecht University (UU).

### Organotypic hippocampal slice preparation and exogenous protein expression

Organotypic hippocampal slices were prepared from Postnatal Day (P)6–8 mice of either sex as described previously (Stoppini et al., 1991) and used at 7–12 d in vitro (DIV) for electrophysiology and 14–21 DIV for imaging. For the expression of exogenous green fluorescent protein (GFP), amyloid precursor protein (APP_CT100_), and GFP- or super ecliptic phluorin (SEP)-tagged rat GluA3 (flip), GluA3_S885A_ and GluA3_K887A_, the respective constructs were cloned into a pSinRep5 shuttle vector. The resulting pSinRep5 plasmids were used to produce infective Sindbis pseudo viruses according to the manufacturer's protocol (Invitrogen B.V.). Sindbis virus infection was achieved by injecting diluted virus into slices 20–52 h prior to the experiments.

### Electrophysiology

During recordings, slices or cultured hippocampal neurons were perfused with artificial cerebrospinal fluid (ACSF) containing the following (in mM): 118 NaCl, 2.5 KCl, 26 NaHCO_3_, and 1 NaH_2_PO_4_, supplemented with 4 MgCl_2_, 4 CaCl_2_, and 20 glucose at 27°C, gassed with 95% O_2_/5% CO_2_. Patch recording pipettes were filled with internal solution containing the following (in mM): 115 CsMeSO_3_, 20 CsCl, 10 HEPES, 2.5 MgCl_2_, 4 Na_2-_ATP, 0.4 Na-GTP, 10 Na-Phosphocreatine, and 0.6 EGTA. Whole-cell recordings were made with 2.1–4.5 MΩ pipettes (*R*_access _< 20 MΩ and *R*_input _> 10 × *R*_access_). During miniature excitatory postsynaptic current (mEPSC) recordings, TTX (1 μM; Tocris Bioscience) and picrotoxin (100 μM; Sigma-Aldrich) were added. During evoked recordings, a cut was made between CA1 and CA3, and picrotoxin (50 μM) was added to the bath. Two stimulating electrodes (two-contact Pt/Ir cluster electrode, Frederick Haer) were placed between 100 and 200 μm down the apical dendrite and 100–300 μm apart laterally. Two neighboring Sindbis-infected and Sindbis-uninfected CA1 neurons were simultaneously recorded. AMPAR-mediated evoked excitatory postsynaptic currents (eEPSCs) were measured as the peak inward current at −60 mV directly after stimulation. Data were acquired using a MultiClamp 700B amplifier (Molecular Devices). Mean EPSC amplitude contained at least 20 sweeps at each holding potential and were acquired using the pClamp 10 software (Molecular Devices). mEPSC data are based on at least 100 events or 10 min of recording and analyzed with MiniAnalysis (Synaptosoft). Individual events above a 5 pA threshold were manually selected by an experimenter blind to the experimental condition.

### Two-photon imaging

3D images were collected by two-photon laser scanning microscopy (Femtonics) with a mode-locked Ti:sapphire laser (Chameleon; Coherent) tuned at 910 nm using a 20× objective. During imaging, slices were kept under constant perfusion of ACSF at 30°C, gassed with 95% O_2_/5% CO_2_. For spine densities, apical dendrites were imaged ∼180 μm from the cell body (pixel size *x*, *y*, *z*: 0.05 × 0.05 × 0.75 μm). The density of spines protruding in the horizontal (*x*/*y*) plane was manually quantified from projections of stacked 3D images by an experimenter blind to experimental condition. For analysis and example images, the intensity value limits of each stacked image were optimized for spine recognition. To monitor the transportation of virally expressed GFP-GluA3 into dendrites, we captured the soma and >150 µm of apical dendrite (pixel size *x*, *y*, *z*: 0.3 × 0.3 × 0.75 μm) using equal microscope settings per condition. For photobleaching experiments, apical dendrites were imaged 150–250 μm from the cell body (pixel size *x*, *y*, *z*: 0.05 × 0.05 × 0.5 μm). Photobleaching of SEP-fluorescence was achieved by prolonged *xy* scanning of isolated spines for 10–20 s, until complete bleaching was visually confirmed (Extended Data Fig. 1-3*A*,*B*). To determine the fluorescence recovery after photobleaching (FRAP), similarly sized *Z*-stacks of dendrites were collapsed for each time point. Background-subtracted green fluorescence of spines was quantified, normalized to that of its dendrite and compared across time. All image analysis was performed with the ImageJ software (fiji.sc).

### Coimmunoprecipitation and immunoblotting

Full-length GluA3 cDNAs were subcloned into a pRK5-Dest vector and GRIP cDNA into a pcDNA3.2-V5-Dest vector. HEK cells were passed 1 d before transfection in DMEM + GlutaMAX (Invitrogen), 10% FBS (Invitrogen), and 1% penicillin–streptomycin (Invitrogen) in 10 cm dishes. Two hours before transfection, the medium of ∼60% confluent cells were refreshed. Cells were transfected with ∼2.5 µg Grip-V5 and GluA3, GluA3_S885A_, or GluA3_K887A_ using PEI 2500. The amount of DNA used for transfection with GluA3 constructs was optimized based on protein expression levels beforehand. After ∼48 h, cells were harvested in 1 ml of a 2% Triton X-100 ice-cold immunoprecipitation buffer (25 mM HEPES/NaOH, 150 mM NaCl), pH7.4, containing 2% Triton X-100 and EDTA-free protease inhibitor cocktail (Roche Diagnostics). The resulting samples were incubated for 1 h at 4°C and spun down twice at 20,800 × *g* for 10 min at 4°C. Anti-Grip (4 µg ABN27, Merck Millipore) was added to the supernatants and incubated overnight at 4°C. The next day, protein A/G PLUS-agarose beads (40 µl; Santa Cruz Biotechnology) were added for 1 h at 4°C and washed four times with immunoprecipitation buffer containing 1% Triton X-100. Proteins were eluted in SDS sample buffer (55 µl), boiled for 5 min, and loaded on a 4–15% Criterion TGX Stain-Free precast gel (Bio-Rad Laboratories). Protein samples were transferred unto a PVDF membrane (Bio-Rad Laboratories) overnight at 40 V. The blots were blocked in 5% milk in TBST and incubated with primary and secondary antibody in 3% milk in TBST. The following antibodies were used: anti-GluA2/3 (1:2,000; CQNFATYKEGYNVYGIESVKI, custom made at GenScript Biotech; Chen et al., 2014) and anti-V5 (1:1,000; ab27671, Abcam) in combination with goat anti-rabbit-HRP (DAKO 1:10,000) and goat anti-mouse-HRP (DAKO, 1:10,000). Membranes were developed using ECL femto (Thermo Fisher Scientific).

### Culturing and transfection of primary hippocampal neurons

Primary hippocampal neuronal cultures were prepared from embryonic day 18 (E18) Janvier Wistar rat brains (either sex) as described in Cunha-Ferreira et al. (2018). Dissociated neurons were plated on coverslips coated with poly-L-lysine (37.5 μg/ml, Sigma-Aldrich) and laminin (1.25 μg/ml, Roche Diagnostics) at a density of 100,000 neurons per well of a 12-well plate. Cultures were plated in 1 ml Neurobasal medium (NB) supplemented with 2% B27 (Invitrogen), 0.5 mM glutamine (Invitrogen), 15.6 μM glutamate (Sigma-Aldrich), and 1% penicillin–streptomycin (Invitrogen) at 37°C in 5% CO_2_. For electrophysiology experiments, dissociated neurons were plated without penicillin–streptomycin (Bahrami and Janahmadi, 2013) and measured at 18–19 DIV, 24 h after exposure to stabilized Aβ oligomers [0.04 µM, Good Biomarker Sciences (GBS) Leiden]. Medium refreshment was done weekly by replacing half of the medium with fresh BrainPhys (Invitrogen) medium supplemented with SM1 (STEMCELL Technologies) and 1% penicillin–streptomycin, supplemented BP medium. At 13–15 DIV, neurons were transfected with various combinations of plasmids to express SEP-GluA3, SEP-GluA3_K887A_, ALFA-GluA3, or Homer1c-GFP under control of a cytomegalovirus promotor or with GFP or Homer1c-ALFA under control of a CaMKII promotor. Before transfection, 0.5 ml medium was transferred from each well to a new culture plate with 0.5 ml fresh BrainPhys (Invitrogen) medium supplemented with SM1 (STEMCELL Technologies) and 1% penicillin–streptomycin. For each well, 1.8∼1–2 μg DNA was mixed with 3.3 μl Lipofectamine 2000 (Invitrogen) in 200 μl NB, incubated for 30 min at RT and added to the neurons. After 2–3 h, coverslips were transferred to the new culture plate and kept at 37°C in 5% CO_2_.

### Antibody feeding procedure

During the antibody feeding procedure, primary hippocampal neuronal cultures were kept at 37°C in 5% CO_2_ in the supplemented BrainPhys medium. At 22 DIV, the neurons were exposed to primary antibodies against SEP (rabbit anti-GFP, 1:2,000, AB_591819) or ALFA (rabbit anti-ALFA, 1:1,000, NanoTag N1581) for 1 h and, 5 h later, were fixed in 4% PFA with 0.1% sucrose for ∼4 min. Stabilized Aβ oligomers (0.04 µM, GBS Leiden) were added ∼24 h before fixation, and leupeptin (100 µg/ml, Sigma-Aldrich) was present during the antibody feeding procedure only. For immunolabeling of surface SEP-GluA3, the neurons were washed three times with phosphate-buffered saline (PBS) supplemented with 100 mM glycine (PBS-gly), blocked with 10% normal goat serum (NGS) in PBS-gly and incubated with anti-rabbit Alexa Fluor 568 (1:500) with 5% NGS in PBS-gly for 1 h. For the experiments using ALFA-GluA3, the surface anti-ALFA antibodies were occupied with goat anti-rabbit Alexa Fluor 405 antibodies to ensure a selective labeling of internalized ALFA antibodies after permeabilization (Extended Data Fig. 6-1). After the occupation of surface ALFA-GluA3, neurons were permeabilized and incubated with Rab7 antibody (ab50533, Abcam) overnight at 4°C. To immunolabel internalized ALFA-GluA3 or SEP-GluA3, the neurons were washed three times with PBS-gly and permeabilized with 0.01% Triton X-100 and 10% NGS in PBS-gly for 30 min and incubated with anti-rabbit Alexa Fluor 647 (1:500) and 5% NGS in PBS-gly for 1 h. During the immunolabeling of internalized ALFA-GluA3, FluoTag-X4 anti-GFP-Alexa Fluor 488 (1:250, NanoTag, N0304) was added to enhance the Homer1c-GFP signal. Finally, neurons were washed three times in PBS-gly and mounted on a glass microscope slide in Mowiol (Sigma-Aldrich).

### Immunohistochemistry on cultured neurons

For the visualization of excitatory synaptic puncta and enhancing GFP and SEP, cultured hippocampal neurons expressing Homer1c-ALFA with GFP, SEP-GluA3, or SEP-GluA3_K887A_ were fixed in 4% PFA with 0.1% sucrose for ∼4 min at 22 DIV. Stabilized Aβ oligomers (0.04 µM, GBS Leiden) were added ∼24 h before fixation. The neurons were washed three times with PBS supplemented with 100 mM glycine (PBS-gly), blocked with 10% NGS in PBS-gly, and incubated with FluoTag anti-ALFA 647N and FluoTag-X4 anti-GFP Atto488 (1:500, NanoTag, N1502 and N0304, respectively) for 1–2 h. Then the neurons were washed three times in PBS-gly and mounted on a glass microscope slide in Fluoromount-G.

### Confocal laser scanning microscopy and quantification

Confocal images were acquired with a Zeiss LSM 700. Oblique dendritic branches of cultured hippocampal neurons were imaged with a 40× oil objective. A *Z*-stack containing 4–7 planes (pixel size *x*, *y*, *z*: 0.1 × 0.1 × 0.7 μm) were acquired, and maximum intensity projections were made for analysis and display. Image analysis was performed in ImageJ. For quantification of synapse density, Homer1c-ALFA puncta were manually quantified on a stretch of ≥20 µm of dendrite. To quantify the levels of surface and internalized GluA3, dendrite and background areas were manually drawn. Background-subtracted surface (S) and internal (I) fluorescent intensities were used to calculate the fraction of internalized SEP-GluA3 [I / (S + I)]. For the colocalization of Rab7 puncta with internalized ALFA-GluA3, Rab7 puncta were manually delineated, and signal intensity of ALFA-GluA3 was measured for each punctum. Next, puncta were discriminated as ALFA-GluA3 negative or positive puncta based on having an ALFA-GluA3 signal three times higher than in the dendritic shaft. Imaging and analysis were done by an experimenter blind to conditions.

### Proteomic analysis

Proteomic analysis was performed as described previously (Vegh et al., 2014). In short, synaptosomes were isolated from hippocampi of APP/PS1-mice and wild-type littermates at 1.5, 3, 6, and 12 months of age as described previously (Li et al., 2007). Five 8-plex isobaric tags that allow relative quantification (iTRAQ) experiments (i.e., five biological replicates per age group per genotype) were performed. Samples were analyzed using an ABI 5800 proteomics analyzer (Applied Biosystems). Protein identification and quantification were performed as described (van Nierop and Loos, 2011). Mascot (Matrix Science, version 2.3.01) searches were performed against SwissProt (version 20/10/2010) and NCBInr (version 20/10/2010) databases. Proteins were considered for quantification if at least three peptides were identified in three replicate iTRAQ sets and at least one peptide in all other sets. Protein abundance was determined by taking the average normalized standardized iTRAQ peak area of all unique peptides annotated to that protein.

### Quantification and statistical analysis

**Figure 1.**
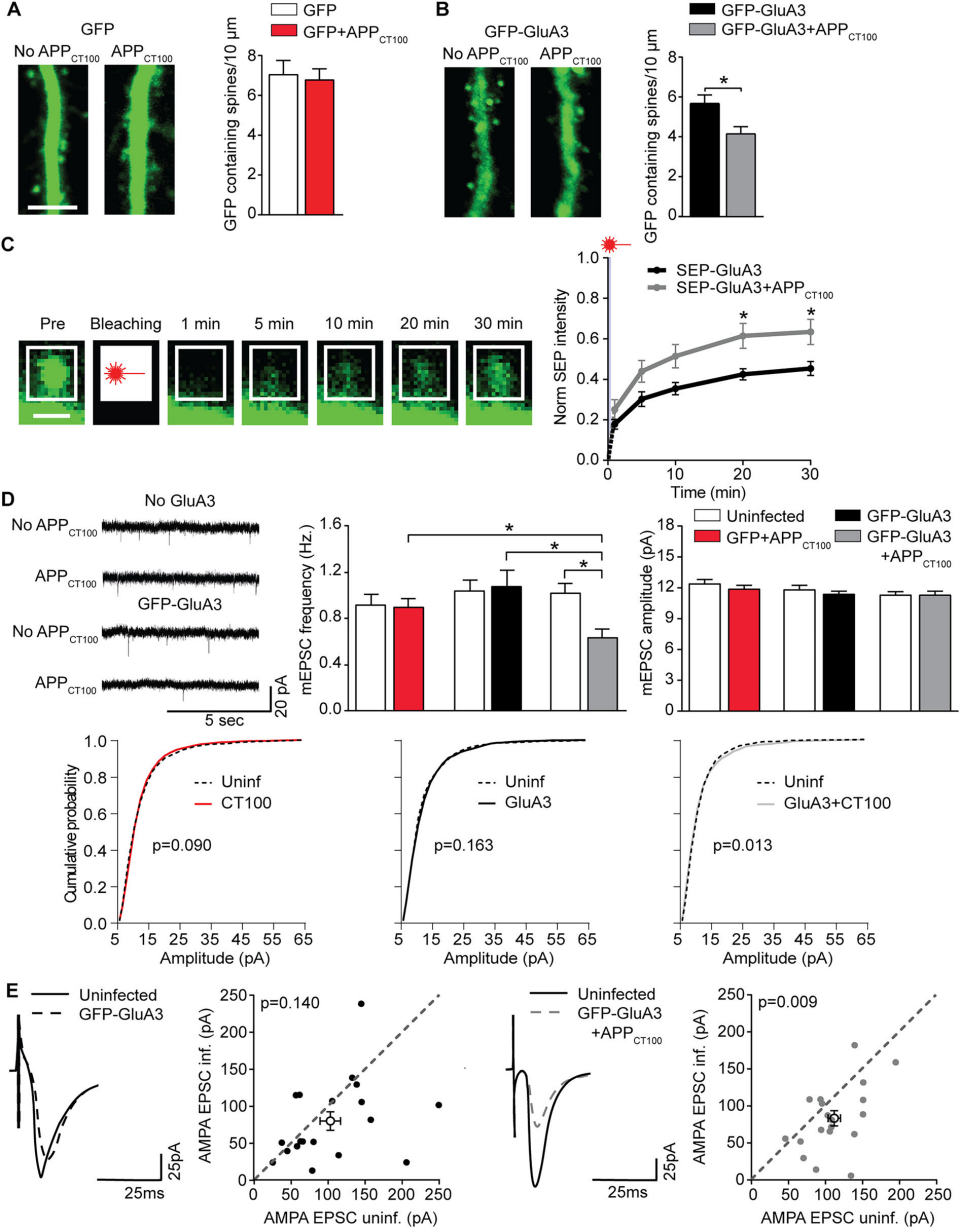
Neuronal expression of GluA3 is sufficient for Aβ to impair synaptic function. Apical dendrites of GluA3-KO neurons expressing GFP (*n* = 19) or GFP + APP_CT100_ (*n* = 15) have similar density of GFP-containing spines. Scale bar, 5 μm. ***B***, GluA3-KO apical dendrites expressing GFP-GluA3 (*n* = 25) or GFP-GluA3 + APP_CT100_ (*n* = 29) showed a different density of GFP-containing spines (*t*_(50)_ = 2.76; *p* = 0.008). Scale bar, 5 μm. See Extended Data Figure 1-2 for more details. ***C***, Left, Time series of the SEP-GluA3-expressing dendritic spine before and after fluorescence bleaching; scale bar, 1 µm. See Extended Data Figure 1-3 for more details. Right, FRAP of dendritic spines expressing SEP-GluA3 (black, *n* = 22) or coexpressing SEP-GluA3 + APP_CT100_ (gray, *n* = 22), demonstrating APP_CT100_ reduced immobile fraction of SEP-GluA3 (*t*_(20)_ and *t*_(30)_, *t*_(84)_ = 2.73 and 2.60; *p* = 0.015). ***D***, (Left) example mEPSC traces, (middle) mEPSC frequency, and (right) mEPSC amplitude of GluA3-KO neurons (uninf. *n* = 24; APP_CT100_ *n* = 29; uninf. *n* = 29; GFP-GluA3 *n* = 30; uninf. *N* = 29; GFP-GluA3 + APP_CT100_ *n* = 27). Only the combined expression of GFP-GluA3 with APP_CT100_ lowered mEPSC frequency (*F* = 3.986; *p* = 0.002; ANOVA, GFP-GluA3 + APPCT100 vs GFP + APP_CT100_ *p* = 0.016; GFP-GluA3 vs GFP-GluA3 + APP_CT100_ *p* = 0.008; uninf. vs GFP-GluA3 + APP_CT100_ *p* = 0.002) but not (right) mEPSC amplitude. Bottom, Cumulative distribution of mEPSC amplitudes (100 events per neuron, *p* values from the K–S test). ***E***, Example traces and dot plots (filled dots represent individual dual recording; open dots denote averages) of simultaneous dual EPSC recordings from neighboring (left; *n* = 18) GFP-GluA3–infected and GFP-GluA3–uninfected GluA3-KO neurons showed no significant synaptic depression unless APP_CT100_ was coexpressed (right; *n* = 19). Data are mean ± SEM. *p < 0.05. Statistics: (***A***, ***B***) unpaired student *t* test; (***C***) unpaired student *t* test with Holm–Šídák multiple-comparison correction; (***D***) one-way ANOVA; (***E***) paired *t* test.

**Figure 2.**
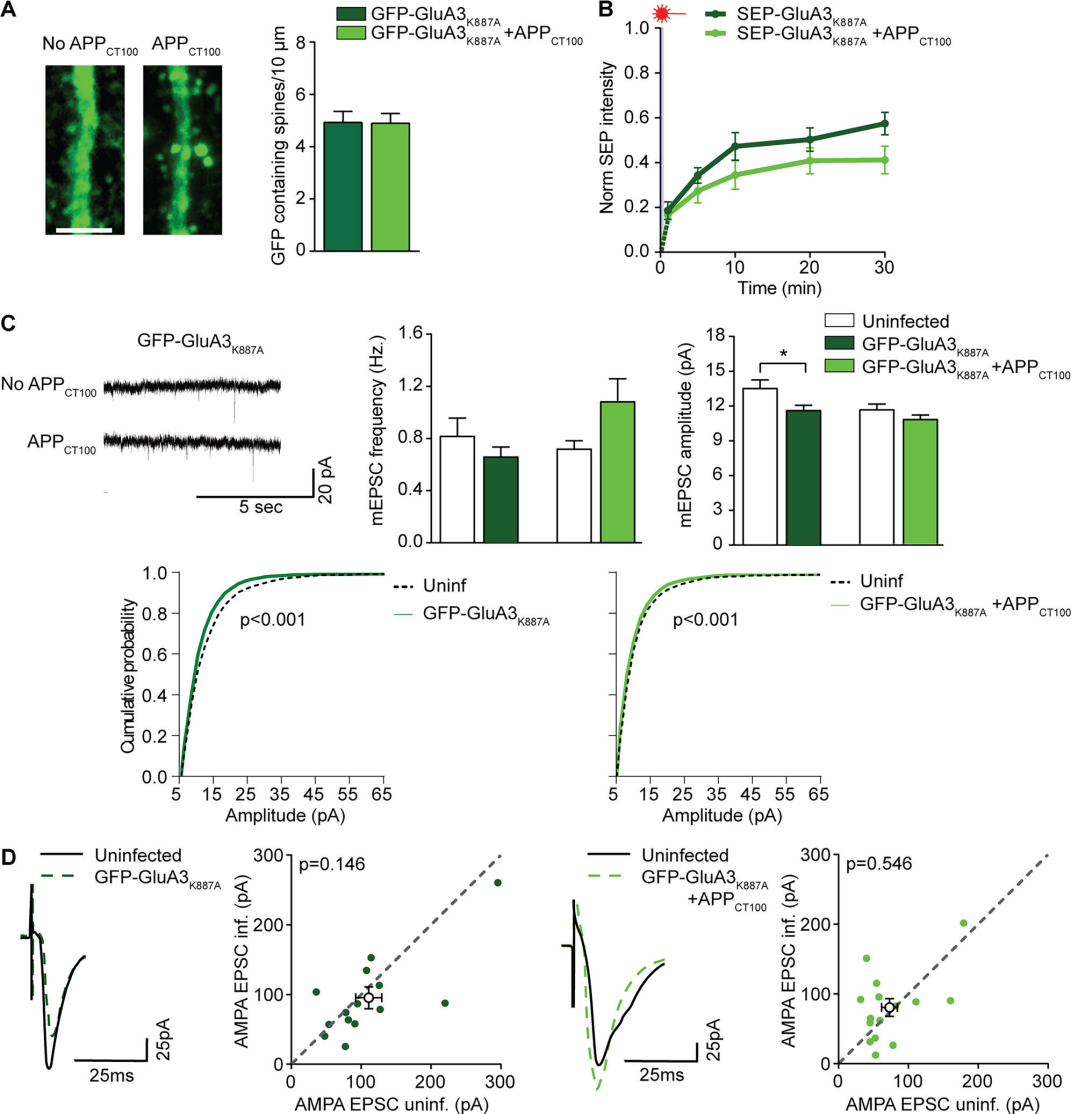
Neuronal expression of GluA3_K887A_ does not sensitize GluA3-KO neurons to Aβ. Density of GFP-containing spines on GFP-GluA3_K887A_ expressing GluA3-KO dendrites was unchanged by APP_CT100_ coexpression (GFP-GluA3_K887A_ *n* = 31; GFP-GluA3_K887A_ + APP_CT100_ *n* = 27; example images scale bar, 5 μm). (***B***, Left) In dendritic GluA3-KO spines expressing SEP-GluA3_K887A_ (dark green, *n* = 12), the coexpression of APP_CT100_ coexpression (light green, *n* = 13) did not affect FRAP. (***C***, Left) Example mEPSC traces of GluA3-KO neurons expressing GFP-GluA3_K887A_ with or without APP_CT100_. Center, Expression of GFP-GluA3_K887A_ with or without APP_CT100_ did not affect mEPSC frequency. Right, GFP-GluA3_K887A_ expression lowered mEPSC amplitude (*t*_(49)_ = 2.27; *p* = 0.028) but not when APP_CT100_ was coexpressed (uninf. *n* = 23; GFP-GluA3_K887A_ *n* = 28; uninf. *n* = 27; GFP-GluA3_K887A _+ APP_CT100_ *n* = 31). Bottom, Cumulative distribution of mEPSC amplitudes (100 events per neuron, *p* values from the K–S test). ***D***, Example traces and dot plots (filled dots represent individual paired recording, open dots denote averages) of (left) paired EPSC recordings from GFP-GluA3_K885A_-expressing GluA3-KO neurons and their uninfected neighbor showed no synaptic depression (*n* = 15), (right) similar to those coexpressing APP_CT100_ (*n* = 14). See Extended Data Figure 2-1 for more details. Data are mean ± SEM. *p < 0.05. Statistics: (***A***) unpaired student *t* test; (***B***) unpaired student *t* test with Holm–Šídák multiple-comparison correction. ***C***, One-way ANOVA; (***D***) paired *t* test.

**Figure 3.**
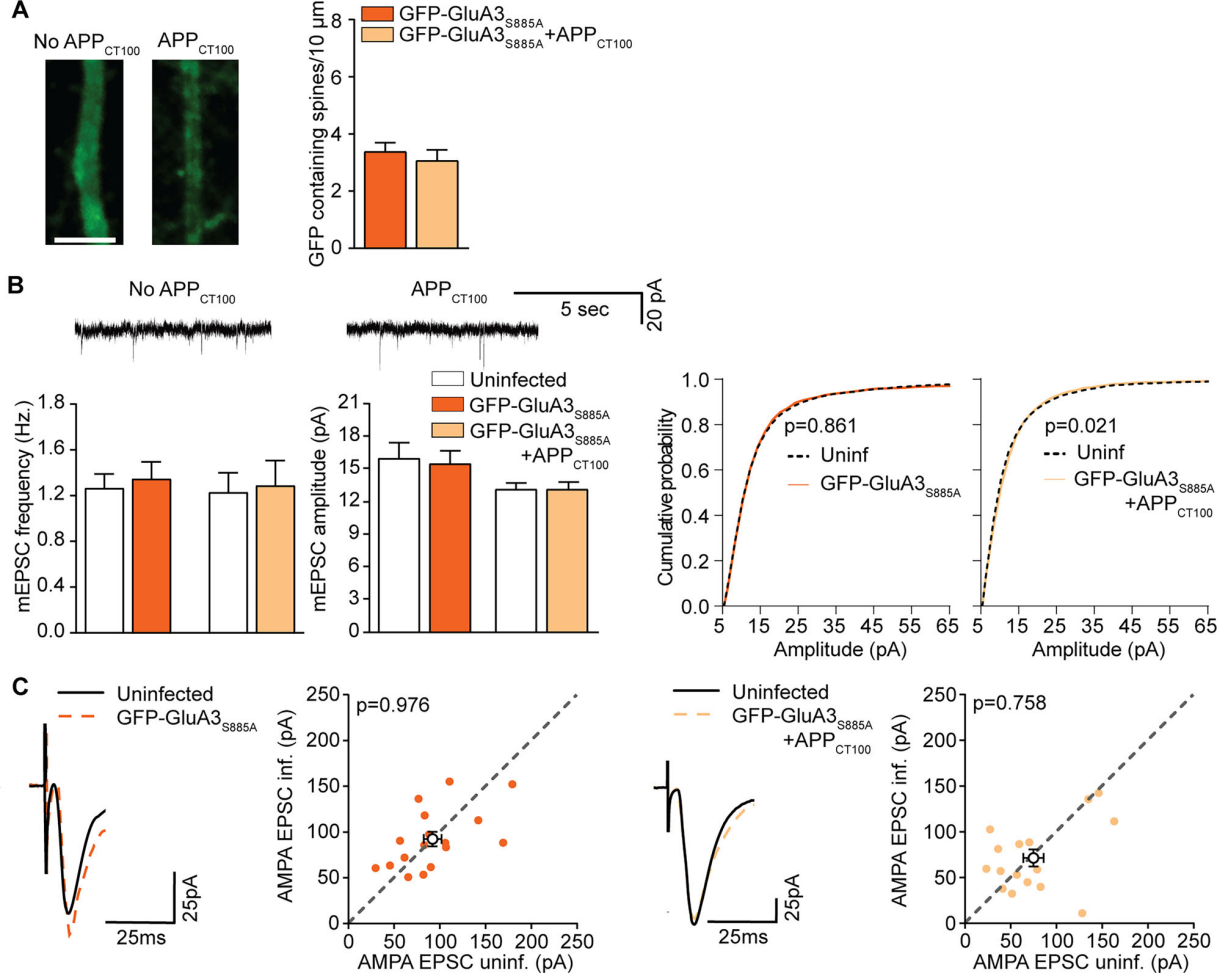
Neuronal expression of GluA3_S885A_ does not sensitize GluA3-KO neurons to Aβ. ***A***, GluA3-KO apical dendrites expressing GFP-GluA3_S885A_ (*n* = 19) or GFP-GluA3_S885A _+ APP_CT100_ (*n* = 17) showed a similar low density of GFP-containing spines. Example images scale bar, 5 μm. ***B***, (Top) example mEPSC traces, (left) mEPSC frequency, and (middle) mEPSC amplitude of GluA3-KO CA1 neurons (uninf. *n* = 22; GFP-GluA3_S885A_ *n* = 23; uninf. *n* = 25; GFP-GluA3_S885A _+ APP_CT100_ *n* = 29) was unaffected by the expression of GFP-GluA3_S887A_ with or without APP_CT100_. Right, Cumulative distribution of mEPSC amplitudes (100 events per neuron, *p* values from the K–S test). ***C***, Example traces and dot plots (filled dots represent individual dual recording; open dots denote averages) of dual EPSC recordings from neighboring infected and uninfected GluA3-KO CA1 neurons expressing GFP-GluA3_S885A_ (left; *n* = 17) or coexpressing GFP-GluA3_S885A _+ APP_CT100_ (right; *n* = 16) showed no synaptic depression. Data are mean ± SEM. Statistics: (***A***) unpaired student *t* test; (***B***) one-way ANOVA; (***C***) paired *t* test.

For each experiment, the desired sample size was based on similar experiments performed previously. *N* represents the number of neurons except for FRAP data where it represents synapses (maximum of three synapses/neuron). Each experiment was repeated in at least three animals per group in organotypic slices or three replicated experiments in cultured neurons. No outliers were excluded. In Figures 1–3 and 5–7, experimental conditions that are depicted in the same graph were performed in parallel and within the same animals. Statistical testing was performed with GraphPad Prism. Where necessary, datasets were log-transformed to obtain normal distributions and homogeneity of variance. Experimental conditions were compared using two-tailed Student’s *t* tests for two conditions (unpaired, unless otherwise indicated) or using one-way ANOVA with post hoc Šídák's multiple-comparison test for more than two conditions. FRAP experiments were analyzed with two-tailed Student’s *t* tests on multiple time points using the Holm–Šídák multiple-comparison correction. Where indicated in the figure captions, two-way ANOVAs or Kolmogorov–Smirnov (K–S) tests were used. *P* values below 0.05 were considered statistically significant.

## Results

### GluA3 expression sensitizes CA1 neurons to Aβ-mediated synaptic depression

To test whether GluA3 expression is required for Aβ-mediated synaptic depression, we virally expressed GFP-GluA3 in a subset of CA1 neurons within organotypic hippocampal slices isolated from GluA3-KO mice. We note that viral infection did not affect the health of CA1 neurons, as assessed by electrophysiological (Extended Data Fig. 1-1), transcriptional, and proteomic profiling (Uyaniker et al., 2019). Forty-eight hours after infection, we imaged GFP-GluA3 fluorescence levels in these CA1 neurons. Consistent with the observation that AMPAR subunits expressed in CA1 neurons by viral transduction accumulate in cell bodies but are transported into dendrites at near-physiological levels (Kessels et al., 2009), fluorescence levels were highest in cell bodies and low in dendrites (Extended Data Fig. 1-2). GFP-GluA3 was detectable at the majority of spines at apical dendrites (Fig. 1*A*,*B*). AMPARs have been shown to *ad libitum* diffuse over the extrasynaptic membrane but to be largely immobilized at synapses (Triller and Choquet, 2005; Ehlers et al., 2007). To measure AMPAR mobility at the spine surface, we tagged GluA3 with the pH-sensitive GFP variant (SEP) to selectively visualize surface receptors (Kopec et al., 2006), and FRAP was analyzed at individual spines (Extended Data Fig. 1-3; Makino and Malinow, 2009). The level of FRAP was ∼45% of the initial spine fluorescence (Fig. 1*C*), indicating that on average 55% of recombinant GluA3 at the spine surface was immobilized and thus considered synaptic. GluA3-containing AMPARs are known to contribute little to basal synaptic currents at CA1 neurons (Renner et al., 2017). Correspondingly, despite the presence of recombinant GluA3 at synapses in the tissue from GluA3-KO mice, such GFP-GluA3 expression did not change synaptic transmission compared with nearby uninfected neurons as measured by mEPSCs (Fig. 1*D*) or measured by eEPSCs obtained by stimulation of Schaffer collateral inputs (Fig. 1*E*).

10.1523/JNEUROSCI.0393-24.2024.f1-1 Figure 1-1 **Sindbis infection does not affect basal membrane resistance of CA1 neurons.** Dual whole-cell patch clamp recordings of input resistance (left) and access resistance (right) of neighboring GluA3-deficient CA1 neurons either infected with GFP-GluA3 or uninfected, 48 hrs after exposure to Sindbis virus. Statistics: unpaired student t-test. Download Figure 1-1, TIF file.

10.1523/JNEUROSCI.0393-24.2024.f1-2 Figure 1-2 **Subcellular distribution of recombinant GFP-GluA3, GFP-GluA3_S885A_ and GFP-GluA3_K887A_ in GluA3-KO CA1 neurons.** (left) The GFP intensity in GluA3-KO CA1 neurons expressing GFP-GluA3, GFP-GluA3_S885A_ and GFP-GluA3_K887A_ is low in dendrites compared to soma (images have equal brightness range, scalebar=10  µm). (right) The ratio of GFP intensity between dendrite and soma is significantly lower in GFP-GluA3_S885A_ (n = 19) expressing neurons compared to those expressing GFP-GluA3 (F = 18.98, p < 0.001; ANOVA; n = 20) or GFP-GluA3_K887A_ (p < 0.001; n = 20). Data are mean ± SEM. *p < 0.001. Statistics: one-way ANOVA. Download Figure 1-2, TIF file.

10.1523/JNEUROSCI.0393-24.2024.f1-3 Figure 1-3 **Extended fluorescent recovery after photo-bleaching data.** (A) SEP signals of spines before and immediately after photo-bleaching demonstrated successful bleaching of SEP fluorescence (n = 26 spines, images have equal brightness range, scale bar: 1  µm). (B) During FRAP experiments, spines nearby photo-bleached areas showed a stable SEP signal during the experiment (SEP-GluA3, black n = 14; SEP-GluA3 + APP_CT100_, grey n = 16; SEP-GluA3_ K887A_, dark green n = 6; SEP-GluA3_K887A_ + APP_CT100_, green n = 12). (C) Fluorescence recovery rate of SEP-GluA3 and SEP-GluA3_K887A_ was unaffected by APP_CT100_ co-expression. Data are mean ± SEM. Download Figure 1-3, TIF file.

We then studied the role of GluA3 in Aβ-mediated synaptic depression by expressing the 100 amino acid long, β-secretase cleavage product of the amyloid precursor protein (APP_CT100_; Maruyama et al., 1990). APP_CT100_ expression produces a loss of synapses and a reduction of postsynaptic currents in remaining synapses (Hsieh et al., 2006; Lumeij et al., 2023) as a consequence of the production of Aβ, most likely of Aβ oligomers (Kamenetz et al., 2003; Wei et al., 2010; Kessels et al., 2013). Whereas APP_CT100_ expression leads to synaptic depression and spine loss in wild-type and GluA1-deficient CA1 neurons, it fails to do so in GluA3-deficient neurons (Fig. 1*A*; Reinders et al., 2016). Upon coexpression of GFP-GluA3 with APP_CT100_ in GluA3-deficient neurons, the number of GFP-containing spines decreased (Fig. 1*B*). FRAP analysis showed that coexpression of APP_CT100_ decreased the immobile fraction of GluA3 at spines by 33% without affecting the recovery rate of mobile GluA3 on the spine surface (Fig. 1*C*; Extended Data Fig. 1-3*C*). Coexpression of APP_CT100_ with GFP-GluA3 caused a significant decrease in mEPSC frequency (Fig. 1*D*; Extended Data Fig. 1-4) and eEPSCs amplitude (Fig. 1*E*). These data indicate that GluA3 expression in neurons lacking GluA3 is sufficient to resensitize these neurons to the damaging effects of Aβ on the functional and structural properties of synapses.

**Figure 4.**
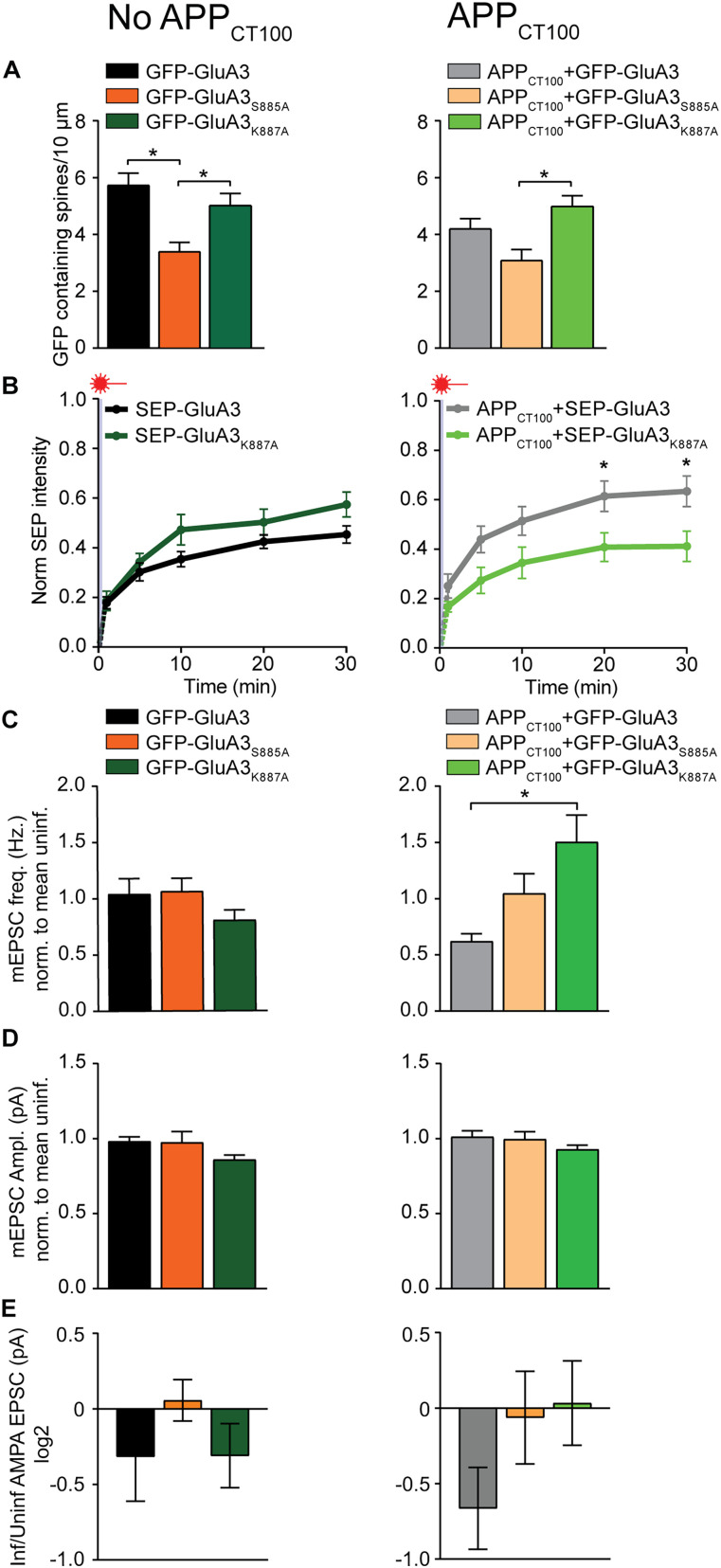
Single amino acid change in GluA3 PDZ motif determines sensitivity of synapses for Aβ. ***A–E***, Comparison of GFP-GluA3 (Fig. 1), GFP-GluA3_S885A_ (Fig. 2), and GFP-GluA3_K887A_ (Fig. 3) without (left) or with coexpression of APP_CT100_. ***A***, Density of GFP-containing spines was lower in GFP-GluA3_S885A_ expressing GluA3-KO dendrites compared with those expressing GFP-GluA3 or GFP-GluA3_K887A_ (*F* = 6.885; *p* = 0.002; ANOVA; left; GFP-GluA3 vs GFP-GluA2_S885A_, *p* = 0.001; GFP-GluA3 vs GFP-GluA3_K887A_, *p* = 0.361; GFP-GluA3_S885A_ vs GFP-GluA3_K887A_, *p* = 0.031), but only compared with GFP-GluA3_K887A_ when APP_CT100_ was coexpressed (*F* = 5.166; *p* = 0.008; ANOVA; right; GFP-GluA3 vs GFP-GluA2_S885A_, *p* = 0.136; GFP-GluA3 vs GFP-GluA3_K887A_, *p* = 0.306; GFP-GluA3_S885A_ vs GFP-GluA3_K887A_, *p* = 0.006). ***B***, FRAP in dendritic GluA3-deficient spines expressing SEP-GluA3 and SEP-GluA3_K887A_ was similar (left; *t*_(20)_, *t*_(32)_ = 1.46; *p* = 0.154; *t*_(30)_, *t*_(32)_ = 2.01; *p* = 0.102) but different with APP_CT100_ coexpression (right; *t*_(20)_, *t*_(33)_ = 2.25; *p* = 0.048 and *t*_(30)_, *t*_(33)_ = 2.36; *p* = 0.048). ***C***, Expression of GFP-GluA3, GFP-GluA3_S885A_, and GFP-GluA3_K887A_ similarly affected mEPSC frequency compared with their uninfected neighbors (left; *F* = 1.348; *p* = 0.266, ANOVA) but with APP_CT100_ coexpression; GFP-GluA3 had a lower mEPSC frequency compared with GFP-GluA3_K887A_ but not GFP-GluA3_S885A_ (right; *F* = 5.393; *p* = 0.006, ANOVA; GFP-GluA3 vs GFP-GluA2_S885A_, *p* = 0.104; GFP-GluA3 vs GFP-GluA3_K887A_, *p* = 0.005; GFP-GluA3_S885A_ vs GFP-GluA3_K887A_, *p* = 0.639). ***D***, Average mEPSC amplitude was unaffected by GFP-GluA3 variants (left; *F* = 2.641; *p* = 0.078; ANOVA), also with APP_CT100_ expression (right; *F* = 0.804; *p* = 0.327; ANOVA). ***E***, Changes in the eEPSC amplitude ratio between infected and uninfected GluA3-deficient neurons were not significantly different (left; *F* = 0.804; *p* = 0.454; ANOVA), also with APP_CT100_ coexpression (right; *F* = 1.840; *p* = 0.170; ANOVA). Data are mean ± SEM. *p < 0.05. Statistics: (***A***, ***C–E***) one-way ANOVA; (***B***) unpaired student *t* test with Holm–Šídák multiple comparison.

10.1523/JNEUROSCI.0393-24.2024.f1-4 Figure 1-4 **Cumulative distribution of mEPSC inter-event intervals .** Cumulative distribution of the time between mEPSCs of (A) APP_CT100_, (B) GFP-GluA3 and (C) GFP-GluA3 + APP_CT100_ expressing neurons compared to uninfected GluA3-deficient neurons. 100 events per neuron, K-S test. Download Figure 1-4, TIF file.

### The GluA3 PDZ-binding motif is required for Aβ-induced synaptic depression

We examined if interactions mediated by the GluA3 C-tail are necessary for GluA3 to sensitize synapses to the effects of Aβ. GluA3 with lysine 887 substituted with alanine (GluA3_K887A_) was generated. This mutation preserves GluA3 interaction with GRIP (Extended Data Fig. 2-1) but prevents AMPAR endocytosis (Kreegipuu et al., 1998; Chung et al., 2000). Upon expression of GFP-GluA3_K887A_ in GluA3-deficient neurons, GFP was detected in dendrites and spines in normal amounts (Fig. 2*A*; Extended Data Fig. 1-2), and FRAP analysis showed that SEP-GluA3_K887A_ and wild-type GluA3 were similarly immobilized at spines (Fig. 2*B*; Extended Data Fig. 1-3). Expression of GFP-GluA3_K887A_ had little effect on synaptic transmission: no effect on eEPSC amplitude or mEPSC frequency, although a small decrease in mEPSC amplitude (Fig. 2*C*,*D*; Extended Data Fig. 2-2). However, in contrast to the expression of wild-type GFP-GluA3 with APP_CT100_, the expression of GFP-GluA3_K887A_ with APP_CT100_ did not cause a loss in GFP-containing spines (Fig. 2*A*), did not reduce the fraction of immobile GluA3_K887A_ (Fig. 2*B*), and did not decrease mEPSC frequency, mEPSC amplitude (Fig. 2*C*), or eEPSC amplitude (Fig. 2*D*). Thus, mutating a single amino acid in the PDZ-binding motif of GluA3 that prevents its endocytosis was sufficient to prevent Aβ-mediated synaptic depression.

10.1523/JNEUROSCI.0393-24.2024.f2-1 Figure 2-1 **GluA3_S885A_ disables GluA3-GRIP interaction.** Western blots from GRIP-V5 IPs on HEK cells expressing a GluA3 variant and/or GRIP-V5. GRIP was immunoprecipitated and stained with an antibody against its fused V5 epitope, GluA3 was stained with anti GluA2/3 antibody. IP = Immunoprecipitation. Download Figure 2-1, TIF file.

10.1523/JNEUROSCI.0393-24.2024.f2-2 Figure 2-2 **Cumulative distribution of mEPSC inter-event intervals in GluA3_K887A_-expressing neurons.** Cumulative distribution of the time between mEPSCs of (A) GFP-GluA3_K887A_ and (B) GFP-GluA3_K887A_ + APP_CT100_ expressing neurons compared to uninfected GluA3-deficient neurons. 100 events per neuron, p-values from K-S test. Download Figure 2-2, TIF file.

The binding of GRIP with GluA2 is known to be necessary for effective trafficking of AMPARs from the cell body to dendrites and their insertion into synapses (Osten et al., 2000; Setou et al., 2002; Heisler et al., 2014; Tan et al., 2015, 2020). Similarly as was previously done for GluA2 (Chung et al., 2000; Osten et al., 2000), we abolished the interaction of GluA3 with GRIP by substituting serine 885 to alanine within its PDZ-binding motif (GluA3_S885A_). We observed that GFP-GluA3_S885A_ fluorescence levels at apical dendrites were substantially lower (Extended Data Fig. 1-2) and as a consequence was detectable at only a small proportion of spines (Fig. 3*A*). Coexpression of GFP-GluA3_S885A_ with APP_CT100_ in GluA3-deficient neurons did not lead to a change in synaptic currents (Fig. 3*B*,*C*; Extended Data Fig. 3-1), indicating that if little GluA3_S885A_ reaches synapses, they are not sensitized to Aβ.

10.1523/JNEUROSCI.0393-24.2024.f3-1 Figure 3-1 **Cumulative distribution of mEPSC inter-event intervals in GluA3_S885A_-expressing neurons.** Cumulative distribution of the time between mEPSCs of (A) GFP-GluA3 **_S885A_** and (B) GFP-GluA3**_S885A_** + APP_CT100_ expressing neurons compared to uninfected GluA3-deficient neurons. 100 events per neuron, p-values from K-S test. Download Figure 3-1, TIF file.

Direct comparison between GFP-GluA3 and single amino acid mutants GFP-GluA3_K887A_ and GFP-GluA3_S885A_ indicates that the K887A mutation did not significantly alter the expression of GluA3 at spines (Fig. 4*A*), the fraction of immobilized GluA3 at spines (Fig. 4*B*), or synaptic currents (Fig. 4*C–E*). However, upon coexpression of APP_CT100_, the K887A mutation fully prevented a decrease in GluA3 immobilized at spines and a depression of synaptic AMPAR currents (Fig. 4*A–E*). These data suggest that Aβ can only trigger synaptic depression when synapses contain GluA3 with competent PDZ–protein interactions.

### Interactions with the GluA3 PDZ-binding motif are required for Aβ oligomers to induce synapse loss

**Figure 5.**
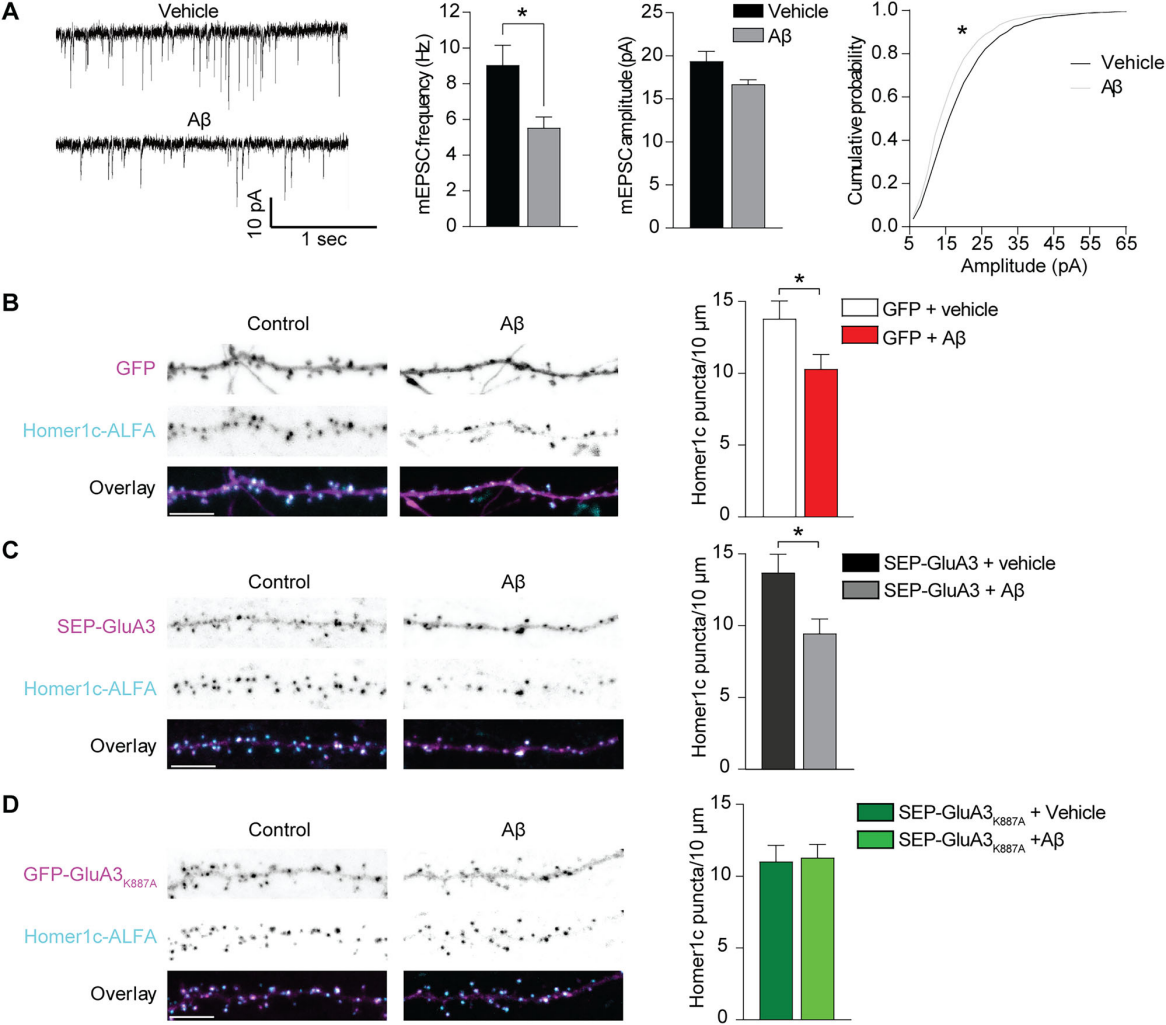
Aβ oligomers trigger synapse loss in cultured neurons that express GluA3 with intact PDZ-binding domain. ***A***, Oligomeric Aβ significantly lowered synaptic currents, as shown in representative example mEPSC traces (left), mEPSC frequency (*t*_(33)_ = 2.42; *p* = 0.021; vehicle *n* = 21; Aβ *n* = 14), average mEPSC amplitude (*t*_(33)_ = 1.84; *p* = 0.076; middle), and mEPSC cumulative distribution in cultured hippocampal neurons (right; 100 event per neuron, *p* values from the K–S test). ***B***, Aβ lowers synapse density on dendrites (GFP, magenta) of cultured hippocampal neurons where synapses are visualized by recombinantly overexpressed Homer1c-ALFA (cyan; *t*_(40)_ = 2.20; *p* = 0.034; control *n* = 20; Aβ *n* = 22). ***C***, Neurons expressing SEP-GluA3 show Aβ-mediated synapse loss (*t*_(55)_ = 2.52; *p* = 0.014; vehicle *n* = 30; Aβ *n* = 27; ***D***) unlike neurons expressing SEP-GluA3_K887A_ (*t*_(48)_ = 0.19; *p* = 0.847; vehicle *n* = 26; Aβ *n* = 24). Data are mean ± SEM. *p < 0.05. Scale bars, 5 μm. Statistics: unpaired student *t* tests.

To investigate whether the presence of GluA3_K887A_-containing AMPARs is sufficient to prevent Aβ-induced synapse loss in a different model system, we used cultured rat hippocampal neurons and exposed them to synthetic Aβ oligomers. In this model system, the presence of Aβ oligomers for 24 h led to synaptic depression as measured by a decrease in mEPSC frequency and a change in mEPSC amplitude distribution (Fig. 5*A*). Correspondingly, Aβ oligomers triggered the loss of excitatory synapses as visualized by expression of the postsynaptic scaffolding protein Homer1c-ALFA (Catsburg et al., 2022; Fig. 5*B*). When coexpressing SEP-GluA3, exposure to Aβ oligomers decreased synapse density (Fig. 5*C*), but not when we coexpressed SEP-GluA3_K887A_ (Fig. 5*D*). In line with our experiments on APP_CT100_-expressing neurons in organotypic slices, these experiments indicate that Aβ oligomers fail to induce a loss of synapses in neurons that express GluA3 with a mutated PDZ-binding domain.

### The GluA3 PDZ motif is required for Aβ-induced lysosomal degradation of GluA3

**Figure 6.**
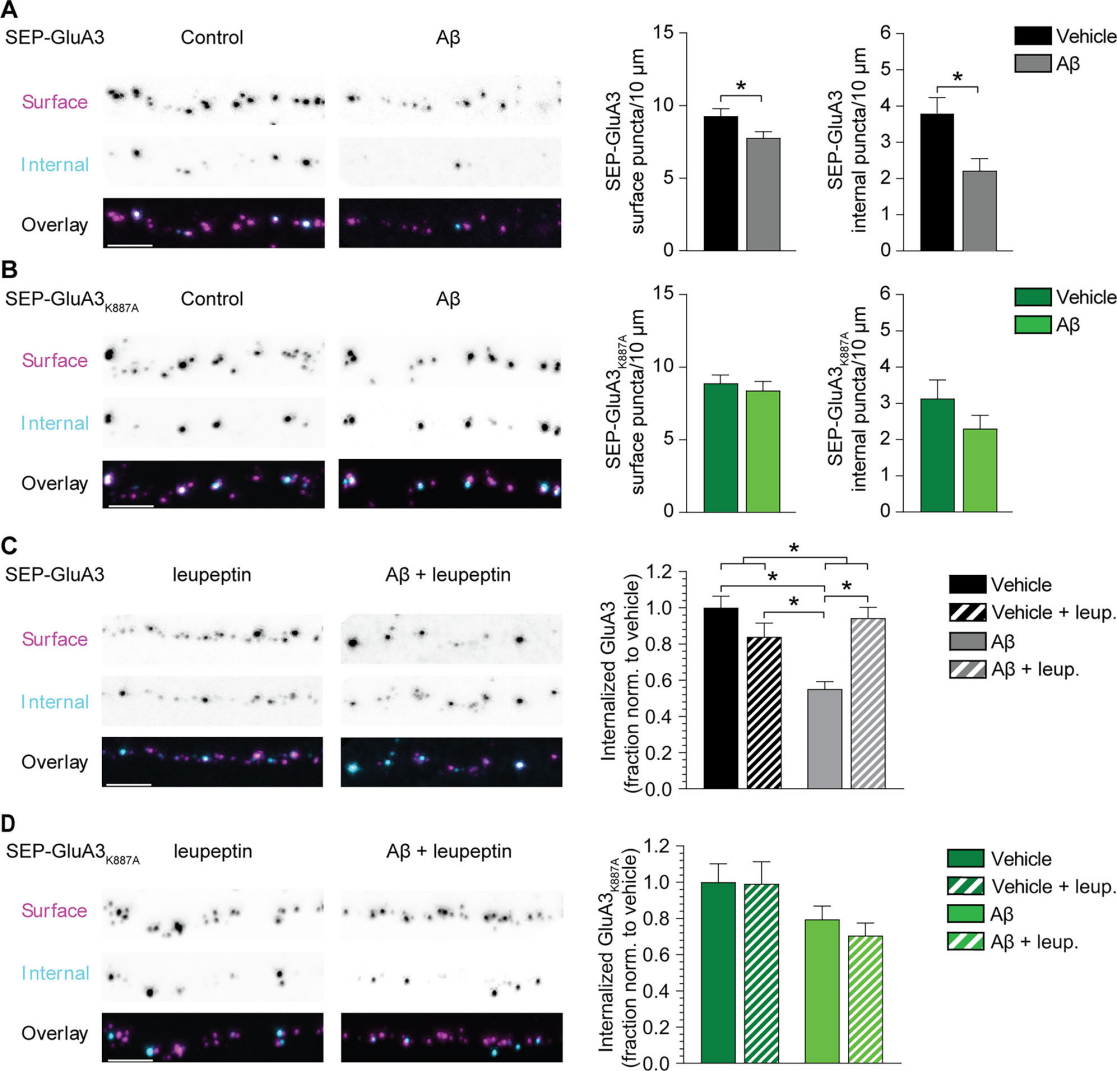
Aβ oligomers induce a loss of surface and internalized GluA3 in cultured neurons. Left, Neurons expressing recombinant (***A***) SEP-GluA3 or (***B***) SEP-GluA3_K887A_ where dendritic surface SEP-GluA3 (magenta) and internalized SEP-GluA3 (cyan) was labeled separately. See Extended Data Figure 6-1 for more details. (***A*** right) Aβ lowered surface (*t*_(73)_ = 2.20; *p* = 0.031) and internalized (*t*_(73)_ = 2.84; *p* = 0.006) SEP-GluA3 puncta (***B*** right) but not surface and internalized SEP-GluA3_K887A_ puncta (SEP-GluA3 puncta, vehicle *n* = 40; Aβ *n* = 37; SEP-GluA3_K887A_ puncta, vehicle *n* = 28; Aβ *n* = 22). ***C***, Aβ reduced the fraction of internalized SEP-GluA3 (*F*_(1,145)_ = 20.39; *p* < 0.001; two-way ANOVA) which was rescued by leupeptin (*p* < 0.001; two-way ANOVA interaction; vehicle *n* = 40; Aβ *n* = 37; vehicle + leup. *n* = 36; Aβ + leup. *n* = 36). ***D***, Aβ reduced the fraction of internalized SEP-GluA3_K887A_ (*F*_(1,92)_ = 6.501; *p* = 0.012; two-way ANOVA). This effect was not altered by leupeptin (vehicle *n* = 28; Aβ *n* = 21; vehicle + leup. *n* = 23; Aβ + leup. *n* = 23). Data are mean ± SEM. *p < 0.05. Scale bars, 5 μm. Statistics: (***A***, ***B***) unpaired student *t* test; K–S test for cumulative distributions, (***C***, ***D***) two-way ANOVA.

Interactions at the PDZ domain of GluA2/3s regulate their trafficking between the neuronal surface membrane and intracellular compartments (Hanley, 2018; Moretto and Passafaro, 2018). We therefore investigated whether Aβ affects the trafficking route of GluA3-containing AMPARs from the dendritic surface into intracellular compartments. We expressed recombinant SEP-GluA3 in cultured rat hippocampal neurons and performed an antibody feeding procedure against SEP, in which the surface and internalized pools of SEP-GluA3 were differentially labeled for quantification (Extended Data Fig. 6-1*A*). During the 5 h postlabeling period, recombinant SEP-GluA3 redistributed between the neuronal surface and intracellular compartments (Fig. 6*A*). Exposure to Aβ oligomers for 24 h reduced the number of both surface and internal GluA3 puncta (Fig. 6*A*). To assess whether the reduction in surface and internal puncta depend on PDZ–protein interactions with the GluA3 C-tail, we repeated this experiment with SEP-GluA3_K887A_. SEP-GluA3_K887A_ puncta distributed similarly between surface and intracellular compartments as SEP-GluA3 puncta (Fig. 6*B*). However, the presence of Aβ oligomers did not significantly affect the number of surface or internal puncta of SEP-GluA3_K887A_ (Fig. 6*B*). These data indicate that Aβ triggers the loss of both surface and internalized SEP-GluA3 via altered protein interactions at its PDZ-binding domain, likely with PICK1.

10.1523/JNEUROSCI.0393-24.2024.f6-1 Figure 6-1 **Detection of internalized GluA3 with antibody feeding assays.** (A) Control experiment showing minimal detection of internalized fraction of SEP-GluA3 without permeabilization. (t(20) = 6.87, p < 0.001; permeabilized n = 16, non-permeabilized n = 6; scale bar: 5  µm)). (B) The fraction of internalized GluA3_K887A_ is lower than that of internalized GluA3 (t(48) = 2.05, p = 0.046, SEP-GluA3 n = 22, SEP-GluA3_K887A_ n = 28). Data are mean ± SEM. *p < 0.05. Statistics: unpaired student t-test. Download Figure 6-1, TIF file.

We next assessed whether a loss of internal SEP-GluA3 could be explained by the lysosomal degradation of internalized GluA3 (Prinkey et al., 2024). Lysosomal protease activity was inhibited by exposing the neuronal cultures to leupeptin during the antibody feeding procedure. Leupeptin did not affect the fraction of internalized SEP-GluA3 in the absence of Aβ oligomers, suggesting that under basal conditions, the majority of GluA3-containing AMPARs cycle between cell surface and intracellular endosomes without being degraded in lysosomes (Fig. 6*C*). However, in the presence of Aβ oligomers, the addition of leupeptin increased the fraction of internalized SEP-GluA3 by 1.7-fold (Fig. 6*C*). Inhibition of lysosomal protease activity with leupeptin did not affect the fraction of internalized SEP-GluA3_K887A_ in both the absence and presence of Aβ oligomers (Fig. 6*D*), although the fraction of internalized SEP-GluA3_K887A_ was somewhat smaller than for SEP-GluA3 (Extended Data Fig. 6-1*B*). These experiments demonstrate that Aβ oligomers trigger the internalization and subsequent lysosomal degradation of internalized GluA3-containing AMPARs through PDZ-mediated interactions at the GluA3 C-tail.

**Figure 7.**
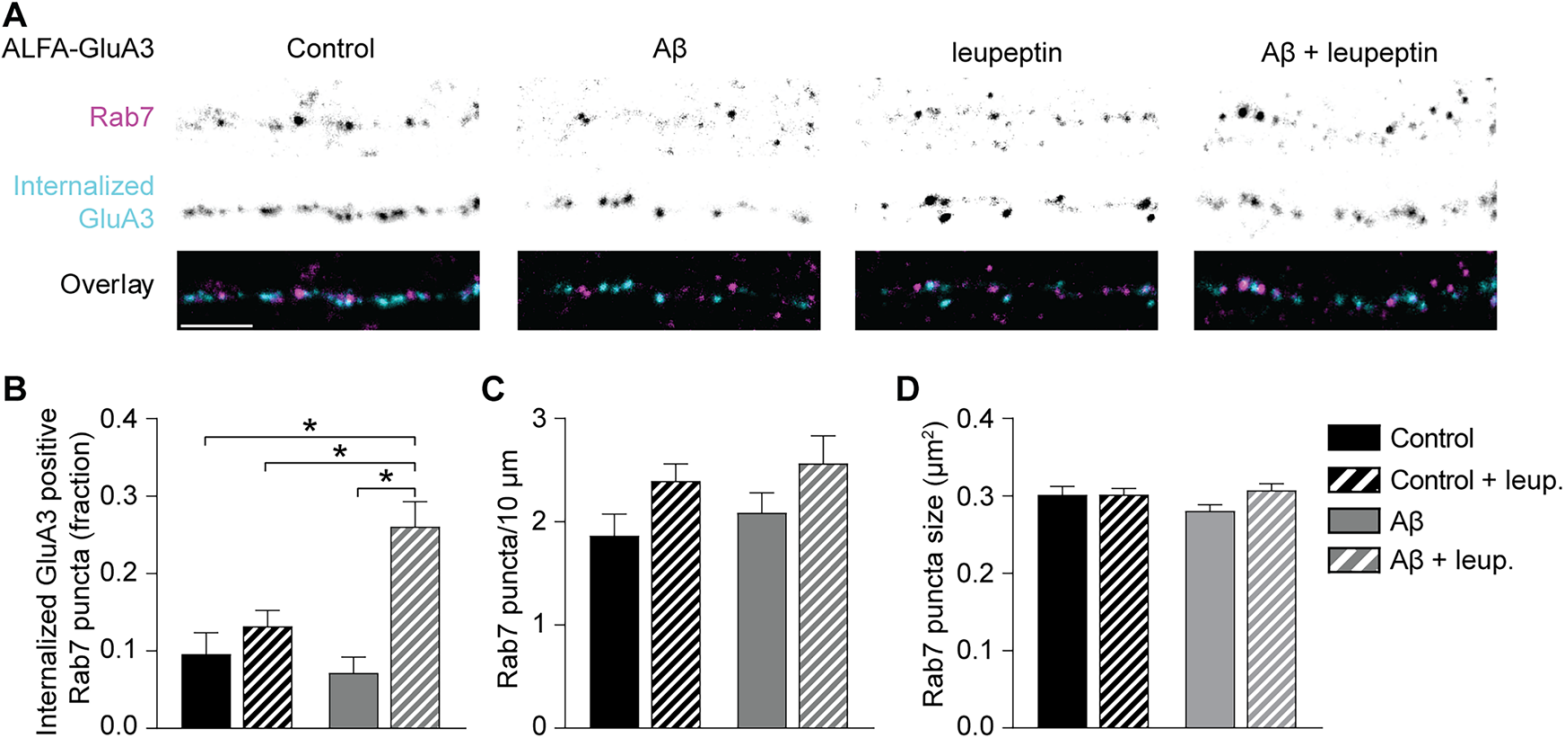
Aβ causes internalized GluA3 to be directed to lysosomes in cultured neurons. ***A***, Dendrites of neurons expressing ALFA-GluA3 where internalized GluA3 (cyan) and endogenous Rab7 (magenta) were labeled. See Extended Data Figure 7-1 for more details. ***B***, Aβ only increased the fraction of internalized GluA3-positive Rab-7 puncta in the presence of leupeptin (*p* < 0.001; two-way ANOVA interaction; Aβ + leup. vs control *p* < 0.001 vs control + leup. *p* = 0.002 vs Aβ *p* < 0.001; control *n* = 22, leup. *n* = 28; Aβ *n* = 26; Aβ + leup. *n* = 22). ***C***, The number of Rab7 puncta was increased by leupeptin (*p* = 0.020; two-way ANOVA) but (***D***) the Rab7 puncta size was unaffected. Data are mean ± SEM. *p < 0.05. Scale bars, 5 μm. Statistics: (***B–D***) two-way ANOVA.

To examine whether internalized GluA3 puncta are indeed localized in endolysosomal compartments upon exposure to Aβ oligomers, we performed the antibody feeding procedure on cultured neurons expressing ALFA-tagged GluA3 and labeled both internalized ALFA-GluA3 and Rab7, a marker for late endosomes and lysosomes (Fig. 7*A*; Bucci et al., 2000; van der Beek et al., 2022). On average ~10% of Rab7 puncta showed enriched levels of internalized ALFA-GluA3 under basal conditions (Fig. 7*B*). Next, we inhibited lysosomal protease activity with leupeptin to make lysosomal ALFA-GluA3 accumulate instead of being degraded. Leupeptin had no effect on the enrichment of Rab7 puncta with ALFA-GluA3 (Fig. 7*B*), indicating that GluA3-containing AMPARs are not degraded in Rab7-positive compartments under basal conditions. However, in the presence of Aβ oligomers, leupeptin increased the fraction of Rab7 puncta enriched with internalized GluA3 by 3.7-fold (Fig. 7*B*), indicating that Aβ oligomers increased the targeting of internalized GluA3 to endolysosomal compartments. This effect was unlikely caused by Aβ oligomer-mediated enlargement of endolysosomal organelles as the number (Fig. 7*C*) and size (Fig. 7*D*) of Rab7 puncta was unaffected. These experiments indicate that in the presence of Aβ oligomers, internalized GluA3-containing AMPARs are targeted to endolysosomal compartments for proteolytic degradation.

10.1523/JNEUROSCI.0393-24.2024.f7-1 Figure 7-1 **Selective labelling of internalized GluA3 in cultured hippocampal neurons after antibody feeding with anti-ALFA antibodies.** Both the blocking after permeabilization (Block all GluA3, cyan), or staining internalized GluA3 without permeabilization (no permeabilization, magenta), effectively minimized the signal of internalized ALFA-GluA3. Scale bar: 5  µm. Data are mean ± SEM. *p < 0.001. Statistics: F(2, 20) = 4.03, p < 0.001, ANOVA. Download Figure 7-1, TIF file.

### GluA3 levels are selectively reduced in 3-month-old APP/PS1-transgenic mice

**Figure 8.**
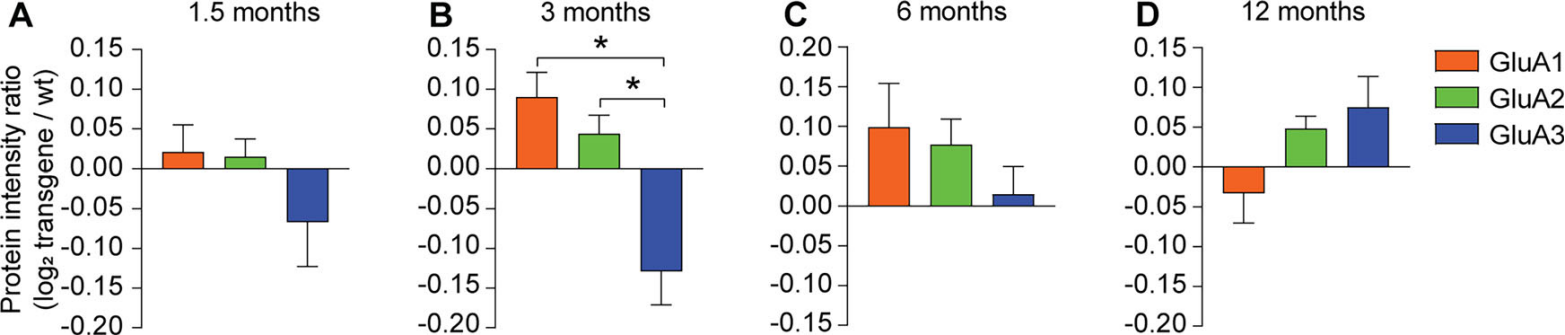
GluA3 levels are decreased in synaptosomes of 3-month-old APP/PS1-transgenic mice. ***A–D***, Protein intensity ratios of GluA1, GluA2, and GluA3 in synaptosomes isolated from hippocampi from APP/PS1-mice and their wild-type littermates of various ages, adopted from Vegh et al. (2014). ***A***, Log-fold changes of GluA3 were similar in 1.5-month-old APP/PS1-mice (***B***) but significantly more reduced than that of GluA1 (*F*_(2,12)_ = 12.17; *p* = 0.002; ANOVA) and GluA2 (*p* = 0.009) in 3-month-old APP/PS1-mice. ***C***, Log-fold changes were not significantly different between GluA1, GluA2, and GluA3 in 6- or (***D***) 12-month-old APP/PS1-mice. ***A–D***, *n* = 5 animals; data are mean ± SEM. *p < 0.05. Statistics: (***A–D***) one-way ANOVA.

Our results suggest that Aβ triggers the endocytosis and degradation of GluA3-containing AMPARs in hippocampal neurons. We wondered whether such a loss of GluA3 could also be observed in the hippocampus of APP/PS1-transgenic mice, a mouse model that accumulates Aβ in the brain (Savonenko et al., 2005). We made use of a previously published proteomics analysis that compared the protein levels at synaptosomes isolated from the hippocampi of wild-type and APP/PS1-transgenic littermate mice at various ages (Vegh et al., 2014). In this mass spectrometry analysis, peptides contain isobaric tags that allow relative quantification (iTRAQ). We quantified the protein levels of GluA1, GluA2, and GluA3 in synaptosomes from APP/PS1-mice relative to those from wild-type littermates. For 1.5-month-old APP/PS1-mice, the age at which Aβ oligomers start to accumulate in the hippocampus (Vegh et al., 2014; Martins et al., 2024), a nonsignificant decrease in GluA3 levels was observed in comparison with GluA1 and GluA2 (Fig. 8*A*). However, for 3-month-old APP/PS1-mice, the age where cognitive impairments arise (Vegh et al., 2014; Reinders et al., 2016), GluA3 levels were decreased significantly more than GluA1 and GluA2 levels in APP/PS1-mice compared with those in the wild-type (Fig. 7*B*). For 6- and 12-month-old APP/PS1-mice, changes in AMPAR subunit protein levels did not significantly differ between GluA1, GluA2, and GluA3 (Fig. 8*C*,*D*). These data show that compared with wild-type littermates, APP/PS1-transgenic mice exhibit a selective reduction in GluA3 levels from synaptosomal fractions at early pathological stages.

## Discussion

We here show that the sensitivity of synapses to Aβ depends on the presence of AMPAR subunit GluA3 at synapses. GluA3-deficient CA1 neurons became sensitive for Aβ-driven synaptic depression after expressing recombinant GFP-GluA3. We further establish that GluA3 is endocytosed and targeted to lysosomes when neurons are exposed to Aβ oligomers. All these effects were critically dependent on the PDZ-binding motif of GluA3. After applying a single amino acid substitution in the GluA3 PDZ-binding motif, Aβ was unable to remove GluA3 from synapses, and synapse loss was fully prevented. Our data indicate that the presence of GluA3 at synapses is both necessary and sufficient for Aβ-mediated synaptic deficits to occur. Correspondingly, APP/PS1-mice show a selective loss of GluA3 at an age coinciding with the initial stages of cognitive decline. Based on our findings, we propose that the Aβ-driven removal of GluA3-containing AMPARs from synapses is a critical early step in AD-related pathogenesis.

We previously established that virally expressed GFP-GluA3 is present at synapses as GluA2/3 heteromers and not as GluA3 homomers (Renner et al., 2017), which is in agreement with the poor ability of GluA3 subunits to form homomeric receptors (Rossmann et al., 2011; Coleman et al., 2016). Although recombinantly expressed GFP-GluA3 was present at synapses, GFP-GluA3 expression did not lead to an increase in synaptic currents. GluA3-containing AMPARs have a low open probability and channel conductance compared with GluA1-containing AMPARs and therefore contribute little to synaptic currents (Gutierrez-Castellanos et al., 2017; Renner et al., 2017). In addition, GluA2/3 containing AMPARs can gradually replace AMPARs at synapses (Shi et al., 2001; McCormack et al., 2006), negating an increase in synaptic strength. Expression of GFP-GluA3 can actually lead to synaptic depression (Shi et al., 2001), possibly as a consequence of low-conductive GluA2/3s replacing high-conductive GluA1/2s at synapses. Our data show that the trafficking and subcellular localization of GluA3-containing AMPARs are controlled by protein interactions at the PDZ-binding motif in the GluA3 C-tail. Interaction between GRIP and the AMPAR C-tail is required for AMPAR transport along dendrites and insertion into synapses (Osten et al., 2000; Setou et al., 2002; Heisler et al., 2014; Tan et al., 2015, 2020), which explains our observation that GluA3_S885A_, a mutant GluA3 that fails to bind GRIP, has low detection at dendrites and spines. When the PDZ motif becomes phosphorylated by PKCα, PICK1 instead of GRIP binds the PDZ motif, leading to the endocytosis and lysosomal degradation of AMPARs (Kim et al., 2001; Perez et al., 2001; Fiuza et al., 2017; Koszegi et al., 2017). We find that a single amino acid mutation (GluA3_K887A_) known to maintain GRIP binding while disabling PICK1 binding, likely by preventing its phosphorylation (Kreegipuu et al., 1998; Chung et al., 2000; Seidenman et al., 2003), did not impair GluA3 expression at dendrites and spines but slightly affected its cycling between surface and internal pools. These findings imply that under basal conditions, PKCα phosphorylation and PICK1 binding to GluA3 are infrequent in cultured neurons and organotypic hippocampal slices.

We here demonstrate that an intact PDZ-binding motif in GluA3 is essential for Aβ to trigger synaptic depression. Our findings are in line with studies showing that Aβ-mediated synapse loss depends on PKCα activity and the ability of PICK1 to interact with the PDZ-binding motif of AMPARs (Hsieh et al., 2006; Alfonso et al., 2014, 2016; Ashourpour et al., 2021). We further demonstrate that Aβ causes internalized GluA3 to be directed and degraded into endolysosomal compartments and establish that this is governed by the PDZ-binding motif in the GluA3 C-tail. This effect was uniquely present in conditions of elevated Aβ and is strikingly similar to a study on oxygen- and glucose-deprived neurons where PICK1 selectively targeted GluA2/3s for endocytosis and lysosomal degradation (Dixon et al., 2009; Koszegi et al., 2017). We successfully blocked Aβ-driven effects on synaptic function by altering GluA3 while leaving the remaining GluA2 subunits of the GluA2/3 heterotetrametric AMPARs unaltered. This is surprising considering the large homology between the C-tail of GluA2 and GluA3 subunits and their shared ability to bind PICK1 (Dong et al., 1997; Srivastava et al., 1998; Xia et al., 1999, 2000; Chung et al., 2000; Daw et al., 2000; Kim et al., 2001; Lin and Huganir, 2007). Possibly, all four AMPAR subunits in a GluA2/3 heteromer need to bind PICK1 to initiate synaptic removal. Alternatively, the GluA1 subunit may inhibit PICK1-mediated AMPAR endocytosis, for instance, by recruiting PSD-95 to synapses, which protects synapses from the effects of Aβ (Dore et al., 2021). It is important to note that besides GluA3-containing AMPARs, also GluA1-containing AMPARs are removed from synapses by Aβ (Hsieh et al., 2006; Alfonso et al., 2014; Guntupalli et al., 2017; Prinkey et al., 2024). However, our data imply that this GluA1 removal can only take place provided that GluA3 at synapses can be endocytosed. It will be interesting to assess how this Aβ-mediated synaptic removal of GluA1 depends on the presence of GluA3. Based on our observations, we propose a model in which Aβ oligomers cause synapse loss by triggering a signaling cascade that leads to phosphorylation of the GluA3 C-tail. This then permits PICK1 to bind and selectively remove GluA2/3-containing AMPARs from synapses to direct them to lysosomes.

Dysfunction of the endolysosomal network, which includes endocytic and autophagic pathways, has been recognized as an early characteristic of AD (Peric and Annaert, 2015; Nixon, 2017). The endolysosomal pathway is involved in the processing, sorting, and turnover of proteins such as AMPARs and APP. Our experiments in cultured neurons demonstrate that the addition of Aβ oligomers induce the lysosomal degradation of GluA3 without affecting the number or size of Rab6-positive late endosomes and lysosomes, suggesting this effect is independent of an enlarged endolysosomal machinery. Dysfunction of the endolysosomal system can be induced by the intracellular accumulation of APP_CT100_ independently on Aβ production (Lauritzen et al., 2016). In our model system, APP_CT100_ expression triggers synaptic deficits through the production of Aβ and most likely the production and excretion of Aβ oligomers (Kamenetz et al., 2003; Wei et al., 2010; Kessels et al., 2013). Our results therefore indicate that Aβ mediates synaptic deficits as a consequence of the endocytosis and lysosomal targeting of GluA3-containing AMPARs, but we did not find supportive evidence that in our model systems this is accompanied by impaired endolysosomal function.

Aβ-mediated synaptic deficits include synapse loss and synaptic depression via the removal of synaptic AMPARs (Hsieh et al., 2006; Alfonso et al., 2014; Guntupalli et al., 2017; Prinkey et al., 2024). This is in line with our quantal analysis of eEPSC recordings which predicted that APP_CT100_ expression in wild-type neurons leads to synaptic depression as a consequence of synapse loss and a decrease in the quantal size of the remaining synapses (Lumeij et al., 2023). However, in our experiments, Aβ-driven synaptic depression is reflected by a decrease in mEPSC frequency but not amplitude. This can be explained by the fact that after synaptic depression, the amplitude of many mEPSCs drop below the 5 pA mEPSC detection threshold. The exclusion of this subset of small mEPSCs from the analysis will lower the mean mEPSC frequency and increase the mean mEPSC amplitude. However, the amplitude of the mEPSCs that are still detected will also be lower after synaptic depression, negating the increase in mean amplitude stemming from the loss of small mEPSCs. This explains how Aβ-driven synaptic depression is not necessarily reflected in the mean mEPSC amplitude.

In this study, we used cultures of organotypic slices and primary hippocampal neuron cultures isolated from immature rodents as a model system, raising reservations about its relevance for AD pathophysiology at advanced age. However, we previously showed that spine loss and memory impairment in APP/PS1-transgenic mice require the presence of GluA3 (Reinders et al., 2016). In addition, we here show that these APP/PS1-transgenic mice show a selective reduction of GluA3 in synaptosomes from the hippocampus at an early age. A loss of GluA3 protein levels in the hippocampus is also observed in another APP-transgenic mouse model (Zhang et al., 2023). In older APP/PS1-mice with progressed Aβ pathology, this reduction in GluA3 was not apparent. Possibly in aged APP/PS1-mice, other events that shape AMPAR levels at synapses are emerging, such as altered network activity (Hijazi et al., 2020). Interestingly, a previous study reported a similar selective loss of GluA3 in hippocampi of APP/PS1-mice that could be overcome by optogenetic stimulation of CA3 neurons (Yang et al., 2021). Furthermore, several studies implicated GluA3 gene transcription and protein expression to be associated with cognitive decline in mild cognitively impaired individuals and AD patients (Armstrong et al., 1994; Yasuda et al., 1995; Armstrong and Ikonomovic, 1996; Carter et al., 2004; Bodily et al., 2016; Hondius et al., 2016; Bereczki et al., 2018; Enache et al., 2020; Medina-Vera et al., 2023). Taken together with our data, GluA3 may be involved in the cognitive decline of AD patients, by sensitizing synapses to the effects of Aβ. Synapse preservation is considered a promising treatment strategy against AD (Jackson et al., 2019; Peng et al., 2022). We propose that targeting protein interactions at the PDZ-binding motif of GluA3 is a potential therapeutic approach to preserve synapses in AD.

## Data Availability

Raw data can be made available on request.
