# Supplementary figures and images for: Amyloid-β-Driven Synaptic Deficits Are Mediated by Synaptic Removal of GluA3-Containing AMPA Receptors
Source: J Neurosci. 2025 Jan 8;45(9):e0393242024. doi: 10.1523/JNEUROSCI.0393-24.2024 (PMC11867010; doi:10.1523/JNEUROSCI.0393-24.2024)

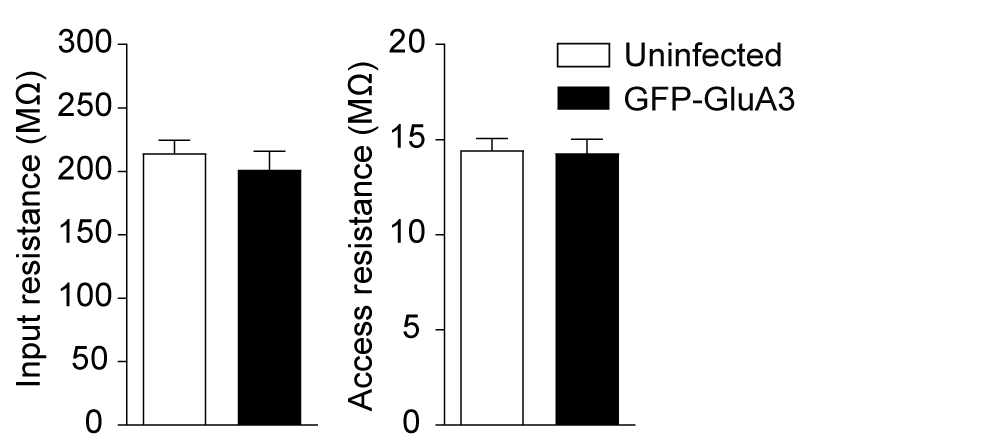

Supplement: Figure 1-1 — Sindbis infection does not affect basal membrane resistance of CA1 neurons. Dual whole-cell patch clamp recordings of input resistance (left) and access resistance (right) of neighboring GluA3-deficient CA1 neurons either infected with GFP-GluA3 or uninfected, 48 hrs after exposure to Sindbis virus. Statistics: unpaired student t-test. Download Figure 1-1, TIF file. [file jneuro-45-e0393242024-s001.tif]

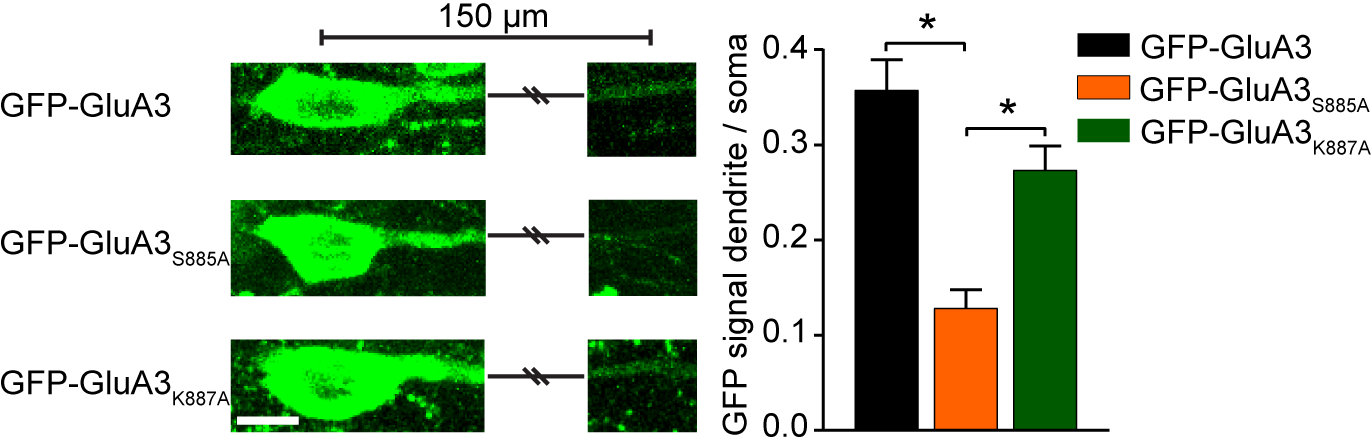

Supplement: Figure 1-2 — Subcellular distribution of recombinant GFP-GluA3, GFP-GluA3S885A and GFP-GluA3K887A in GluA3-KO CA1 neurons. (left) The GFP intensity in GluA3-KO CA1 neurons expressing GFP-GluA3, GFP-GluA3S885A and GFP-GluA3K887A is low in dendrites compared to soma (images have equal brightness range, scalebar=10 µm). (right) The ratio of GFP intensity between dendrite and soma is significantly lower in GFP-GluA3S885A (n = 19) expressing neurons compared to those expressing GFP-GluA3 (F = 18.98, p < 0.001; ANOVA; n = 20) or GFP-GluA3K887A (p < 0.001; n = 20). Data are mean ± SEM. *p < 0.001. Statistics: one-way ANOVA. Download Figure 1-2, TIF file. [file jneuro-45-e0393242024-s002.tif]

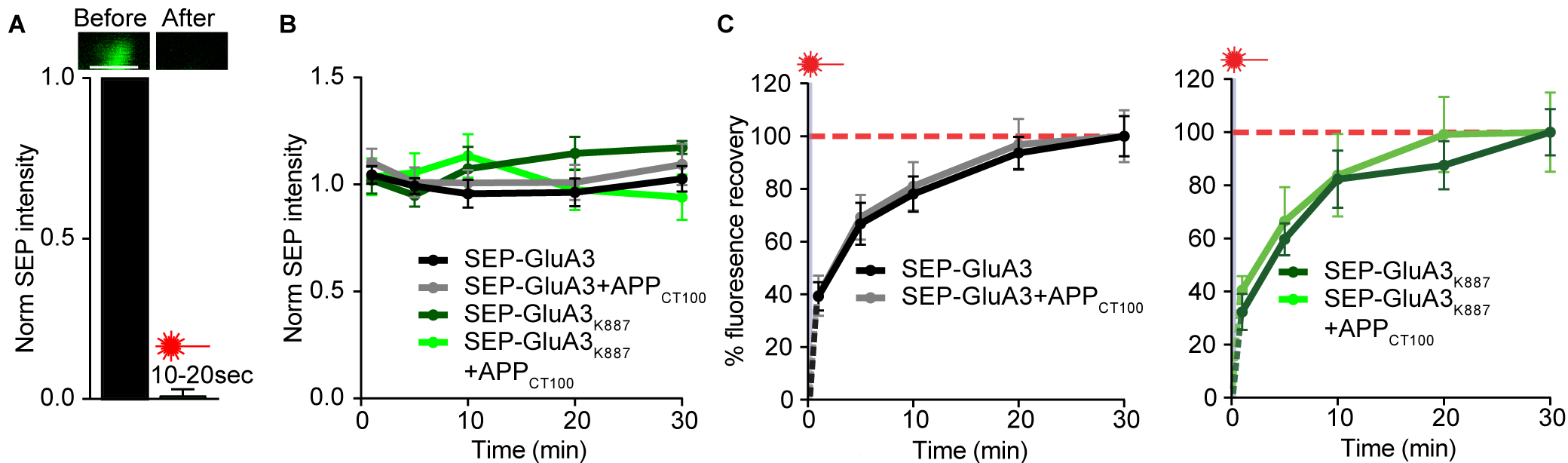

Supplement: Figure 1-3 — Extended fluorescent recovery after photo-bleaching data. (A) SEP signals of spines before and immediately after photo-bleaching demonstrated successful bleaching of SEP fluorescence (n = 26 spines, images have equal brightness range, scale bar: 1 µm). (B) During FRAP experiments, spines nearby photo-bleached areas showed a stable SEP signal during the experiment (SEP-GluA3, black n = 14; SEP-GluA3 + APPCT100, grey n = 16; SEP-GluA3 K887A, dark green n = 6; SEP-GluA3K887A + APPCT100, green n = 12). (C) Fluorescence recovery rate of SEP-GluA3 and SEP-GluA3K887A was unaffected by APPCT100 co-expression. Data are mean ± SEM. Download Figure 1-3, TIF file. [file jneuro-45-e0393242024-s003.tif]

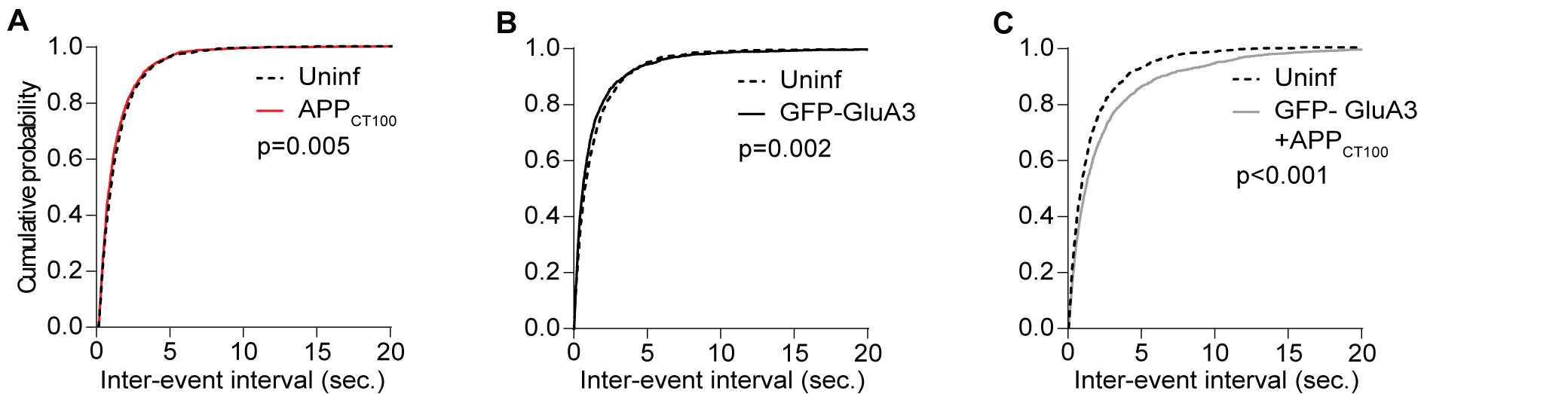

Supplement: Figure 1-4 — Cumulative distribution of mEPSC inter-event intervals . Cumulative distribution of the time between mEPSCs of (A) APPCT100, (B) GFP-GluA3 and (C) GFP-GluA3 + APPCT100 expressing neurons compared to uninfected GluA3-deficient neurons. 100 events per neuron, K-S test. Download Figure 1-4, TIF file. [file jneuro-45-e0393242024-s004.tif]

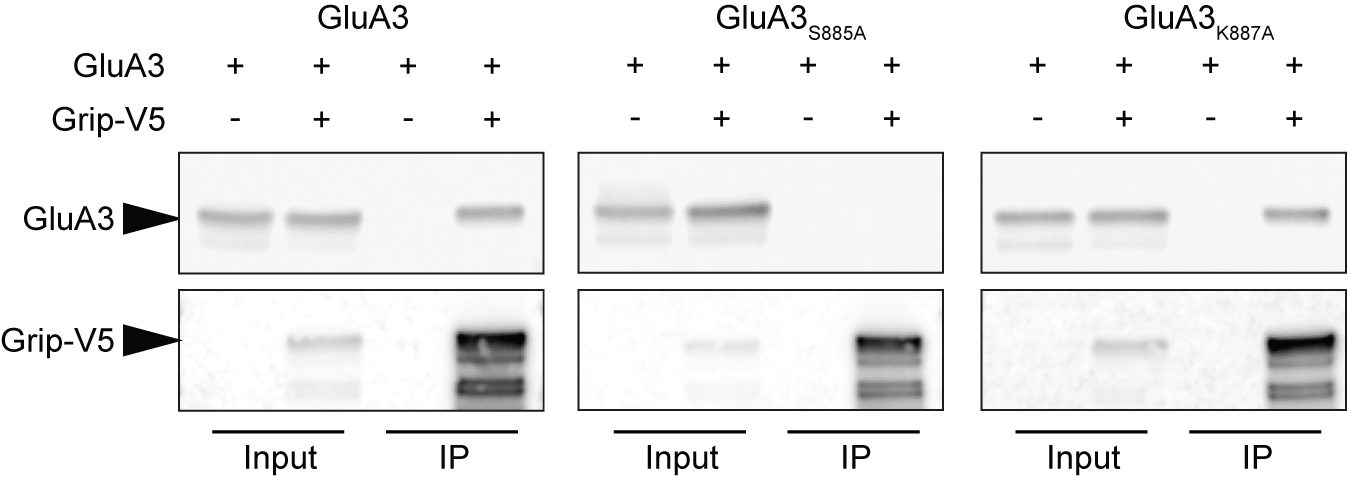

Supplement: Figure 2-1 — GluA3S885A disables GluA3-GRIP interaction. Western blots from GRIP-V5 IPs on HEK cells expressing a GluA3 variant and/or GRIP-V5. GRIP was immunoprecipitated and stained with an antibody against its fused V5 epitope, GluA3 was stained with anti GluA2/3 antibody. IP = Immunoprecipitation. Download Figure 2-1, TIF file. [file jneuro-45-e0393242024-s005.tif]

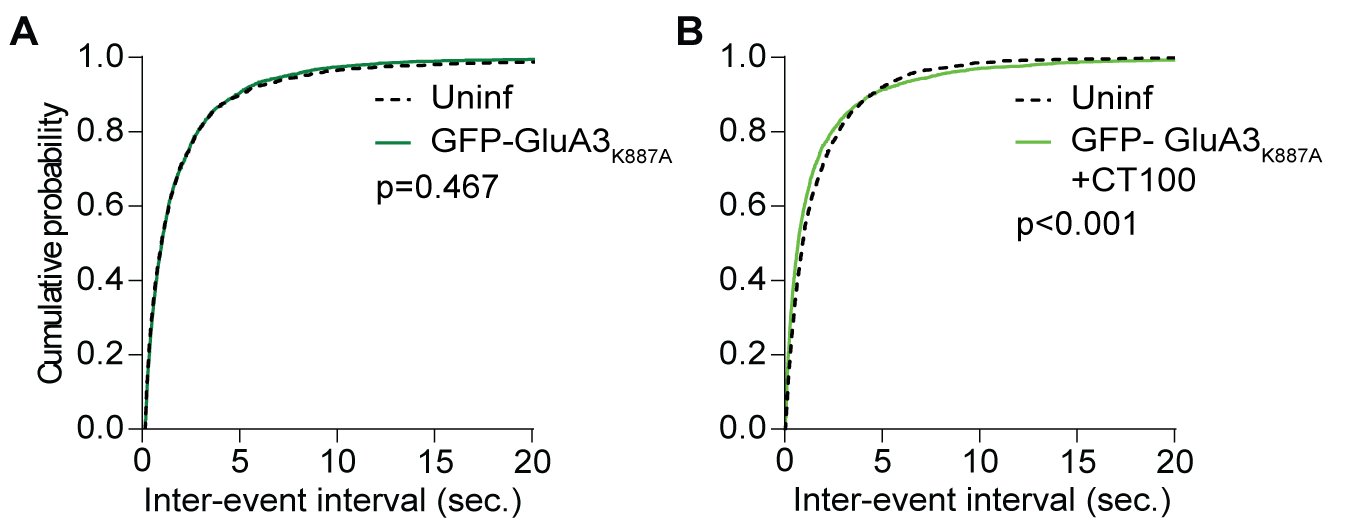

Supplement: Figure 2-2 — Cumulative distribution of mEPSC inter-event intervals in GluA3K887A-expressing neurons. Cumulative distribution of the time between mEPSCs of (A) GFP-GluA3K887A and (B) GFP-GluA3K887A + APPCT100 expressing neurons compared to uninfected GluA3-deficient neurons. 100 events per neuron, p-values from K-S test. Download Figure 2-2, TIF file. [file jneuro-45-e0393242024-s009.tif]

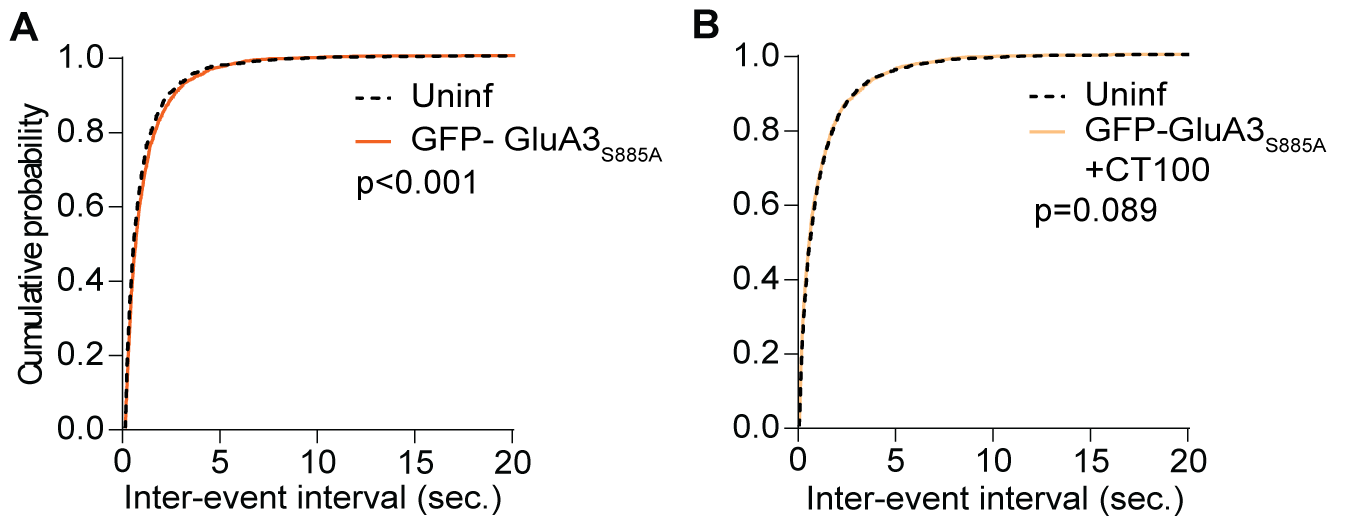

Supplement: Figure 3-1 — Cumulative distribution of mEPSC inter-event intervals in GluA3S885A-expressing neurons. Cumulative distribution of the time between mEPSCs of (A) GFP-GluA3 S885A and (B) GFP-GluA3S885A + APPCT100 expressing neurons compared to uninfected GluA3-deficient neurons. 100 events per neuron, p-values from K-S test. Download Figure 3-1, TIF file. [file jneuro-45-e0393242024-s006.tif]

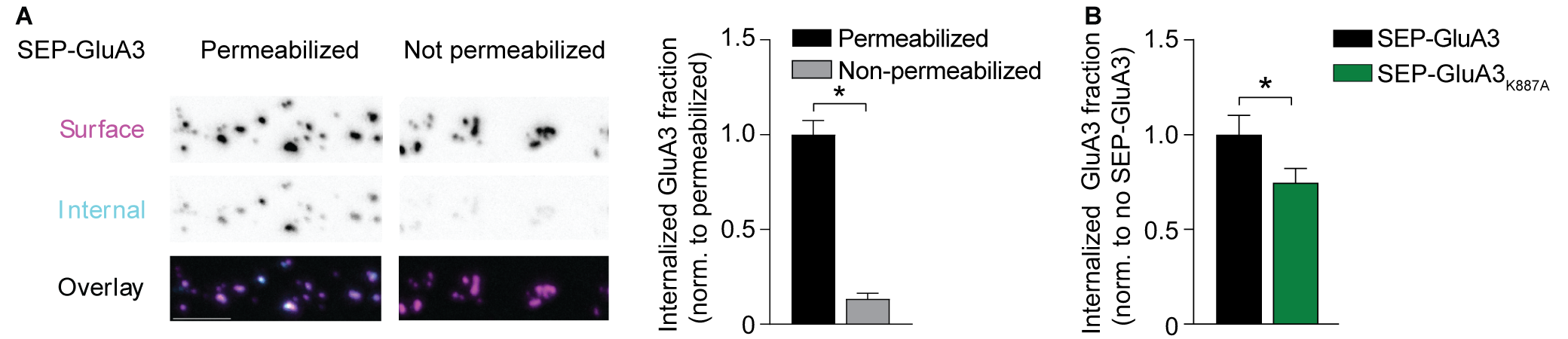

Supplement: Figure 6-1 — Detection of internalized GluA3 with antibody feeding assays. (A) Control experiment showing minimal detection of internalized fraction of SEP-GluA3 without permeabilization. (t(20) = 6.87, p < 0.001; permeabilized n = 16, non-permeabilized n = 6; scale bar: 5 µm)). (B) The fraction of internalized GluA3K887A is lower than that of internalized GluA3 (t(48) = 2.05, p = 0.046, SEP-GluA3 n = 22, SEP-GluA3K887A n = 28). Data are mean ± SEM. *p < 0.05. Statistics: unpaired student t-test. Download Figure 6-1, TIF file. [file jneuro-45-e0393242024-s007.tif]

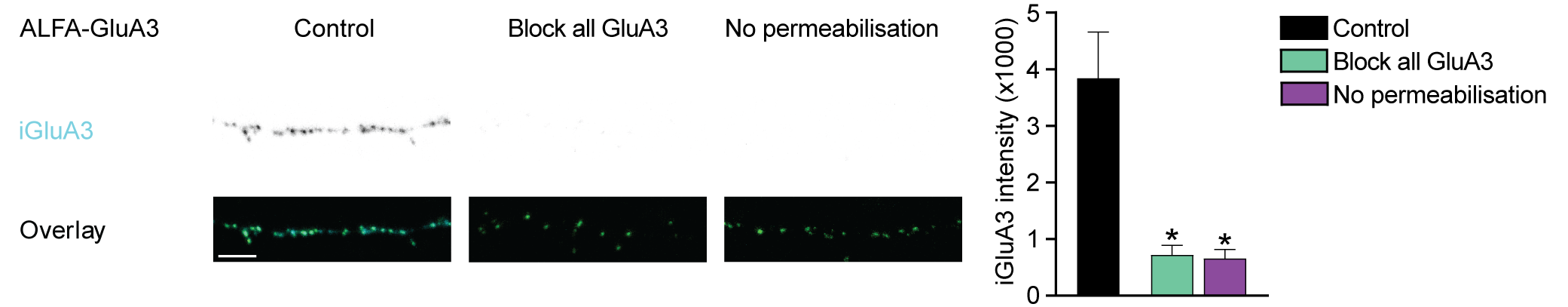

Supplement: Figure 7-1 — Selective labelling of internalized GluA3 in cultured hippocampal neurons after antibody feeding with anti-ALFA antibodies. Both the blocking after permeabilization (Block all GluA3, cyan), or staining internalized GluA3 without permeabilization (no permeabilization, magenta), effectively minimized the signal of internalized ALFA-GluA3. Scale bar: 5 µm. Data are mean ± SEM. *p < 0.001. Statistics: F(2, 20) = 4.03, p < 0.001, ANOVA. Download Figure 7-1, TIF file. [file jneuro-45-e0393242024-s008.tif]
